# Individualistic values are related to an increase in the outbreaks of infectious diseases and zoonotic diseases

**DOI:** 10.1038/s41598-018-22014-4

**Published:** 2018-03-01

**Authors:** Serge Morand, Bruno A. Walther

**Affiliations:** 1 IRAD, UMR ASTRE, F-34398 Montpellier, France; 2 CNRS – Institut des Sciences de l’Evolution de Montpellier, Université de Montpellier, Montpellier, France; 3 Faculty of Veterinary Technology, Kasetsart University, Bangkok, Thailand; 4 Master Program in Global Health and Development, College of Public Health, Taipei Medical University, 250 Wu-Hsing St, Taipei, 110 Taiwan

## Abstract

Collectivist versus individualistic values are important attributes of intercultural variation. Collectivist values favour in-group members over out-group members and may have evolved to protect in-group members against pathogen transmission. As predicted by the pathogen stress theory of cultural values, more collectivist countries are associated with a higher historical pathogen burden. However, if lifestyles of collectivist countries indeed function as a social defence which decreases pathogen transmission, then these countries should also have experienced fewer disease outbreaks in recent times. We tested this novel hypothesis by correlating the values of collectivism-individualism for 66 countries against their historical pathogen burden, recent number of infectious disease outbreaks and zoonotic disease outbreaks and emerging infectious disease events, and four potentially confounding variables. We confirmed the previously established negative relationship between individualism and historical pathogen burden with new data. While we did not find a correlation for emerging infectious disease events, we found significant positive correlations between individualism and the number of infectious disease outbreaks and zoonotic disease outbreaks. Therefore, one possible cost for individualistic cultures may be their higher susceptibility to disease outbreaks. We support further studies into the exact protective behaviours and mechanisms of collectivist societies which may inhibit disease outbreaks.

## Introduction

Numerous studies and reviews have addressed the question of why countries and cultures differ in their tendency to prefer collectivist versus individualistic values and lifestyles^1^. Collectivist values are characterized by moral frameworks and social behaviours which emphasize the group and its interests and therefore favour in-group interests (such as communal, societal, or national interests) over the interests of its individual members, and further favour the interests of in-group members over those of out-group members. Individualistic values, on the other hand, favour the interests of the individuals over the interests of in-group as well as out-group members; they therefore value the independence, self-reliance and self-realization of the individual over communal, societal, or national interests^2^.

In order to explain the cultural variation in this collectivism-individualism continuum, Fincher *et al*.^3^ proposed that the ecological and epidemiological pressures exerted by infectious diseases on the social behaviour of human host populations may partly explain the observed differences in collectivism versus individualism. Their underlying assumption is that collectivist societies, by the nature of their social behaviours, decrease the transmission of pathogens more effectively than individualistic societies do. In other words, collectivist behaviours are hypothesized to generate a societal defence against pathogen transmission. Two mechanisms have been proposed which may underpin this defence function of collectivist societies^3^: first, collectivists are more wary of contact with foreigners and other out-group members, and this ‘xenophobic’ attitude consequently decreases exposure to pathogens^4^. Second, collectivist values enforce conformity and tradition which may also decrease pathogen exposure, e.g., through traditional food preparation methods which decrease pathogen transmission^5^. Collectivist values could thus be considered as one component of human’s social immune system^6^. Since collectivist behaviours are assumed to decrease pathogen transmission, such behaviours should therefore be more likely found in geographical regions in which human societies are exposed to higher pathogen burdens.

For these reasons, Fincher *et al*.^3^ hypothesized that collectivist values should be more prevalent in cultures which were exposed to a higher historical pathogen burden. Using four different measures of the collectivism-individualism continuum and two measures of historical pathogen burden, they confirmed that pathogen burden was positively correlated with collectivist values across countries. Their findings were later supported by other cross-cultural studies which considered other cultural values^7,8^. Thornhill and Fincher^7^ later considered the original hypothesis of Fincher *et al*.^3^ to be a part of their more inclusive “parasite-stress theory of values and sociality” which explains how collectivist values may act as a social defence against pathogen transmission.

We here propose that their overall theory should have another implication which has not been previously tested, to the best of our knowledge. We hypothesize that, since individualistic societies should be more susceptible to disease transmission, they should therefore also be subject to a higher number of infectious disease outbreaks and zoonotic disease outbreaks, including emerging infectious disease events, in recent times. On the other hand, collectivist societies, having maintained their ‘protective’ behaviours, should be less often afflicted by disease outbreaks and epidemics, over the last few decades. Thus, collectivist values should to this day maintain a protective mechanism against the occurrence of disease outbreaks and epidemics.

We investigated this novel hypothesis by using data from outbreaks of infectious diseases and zoonotic diseases that occurred over the last several decades as well as recorded events of emerging infectious diseases documented in Jones *et al*.’s global analysis^9^. We first re-tested the original hypothesis that collectivist values characterize countries with a high contemporary and historical pathogen burden using the historical data compiled by Murray and Schaller^10^ which was not part of Fincher *et al*.’s^3^ original analysis. Second, we tested our novel hypothesis that individualistic countries should be more prone to infectious disease outbreaks, zoonotic disease outbreaks, and emerging infectious disease events. Finally, we used a combination of machine learning and multiple regression techniques to discern which of our independent variables best account for the variation in the collectivism-individualism continuum.

## Results

### Eliminating independent variables and conducting simple correlations

Our first strategy was to eliminate independent variables which clearly explained very little of the variation in the dependent variable Individualism. Elimination of these variables reduced (1) the possibility of multicollinearity among the independent variables, (2) the possibility of overfitting of the statistical model, and (3) the number of all possible models which needed to be tested (cf. part 3 of Results below).

**Figure 1:**
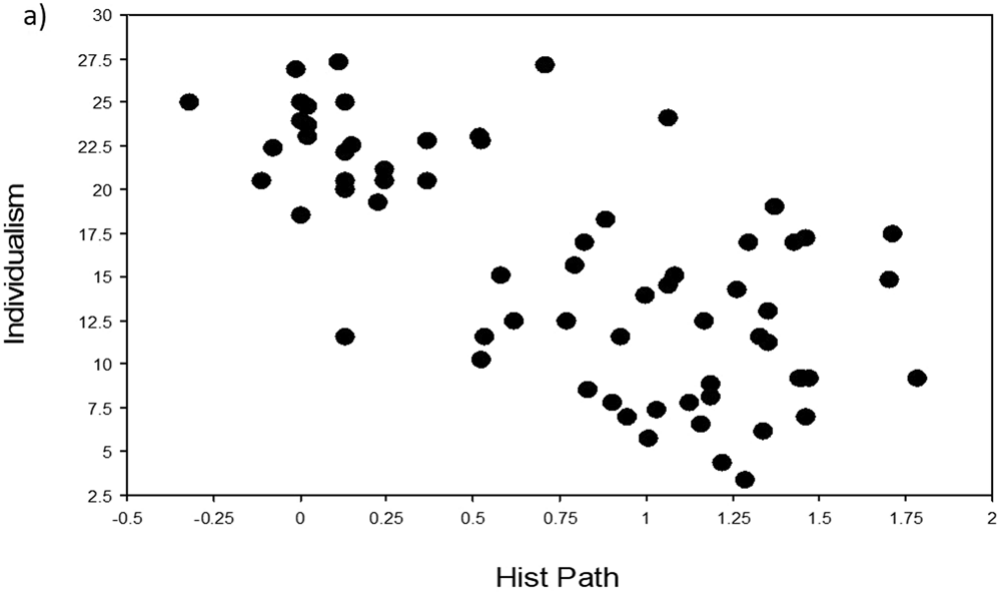
Relationships of Individualism to Hist Path (historical pathogen burden). Scatterplot of Individualism (values of collectivism-individualism for n = 66 countries) against the independent variable Hist Path (variables were Box-Cox transformed, raw data in Supplementary Table S1).

**Figure 2:**
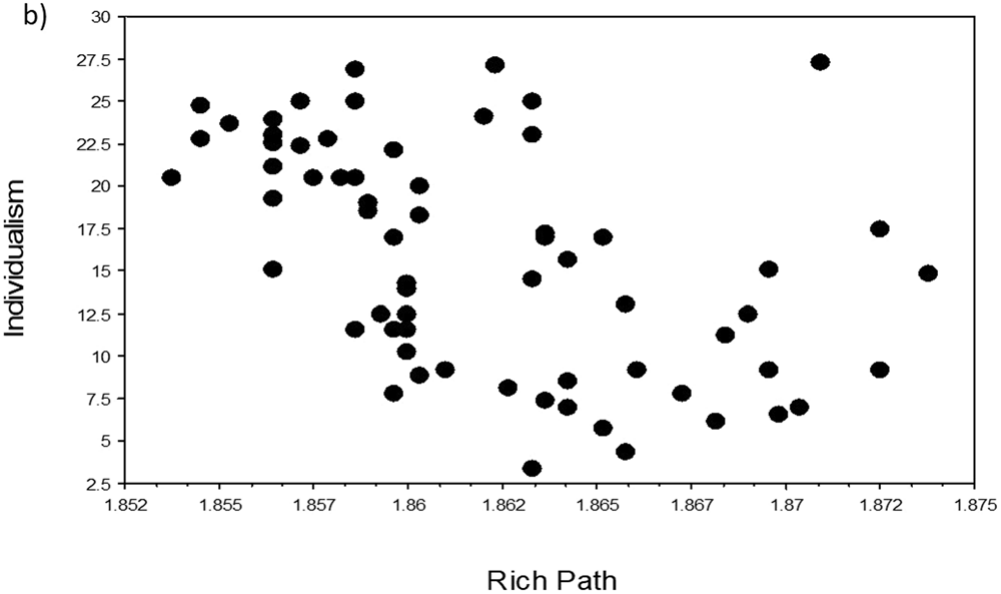
Relationships of Individualism to Rich Path (pathogen richness). Scatterplot of Individualism (values of collectivism-individualism for n = 66 countries) against the independent variable Rich Path (variables were Box-Cox transformed, raw data in Supplementary Table S1).

**Figure 3:**
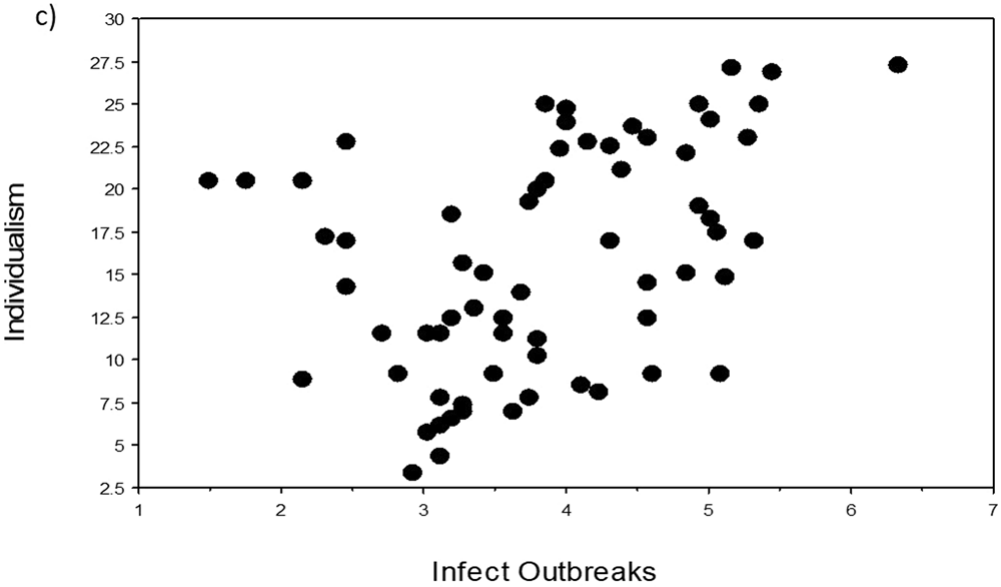
Relationships of Individualism to Infect Outbreaks (number of infectious disease outbreaks). Scatterplot of Individualism (values of collectivism-individualism for n = 66 countries) against the independent variable Infect Outbreaks (variables were Box-Cox transformed, raw data in Supplementary Table S1).

**Figure 4:**
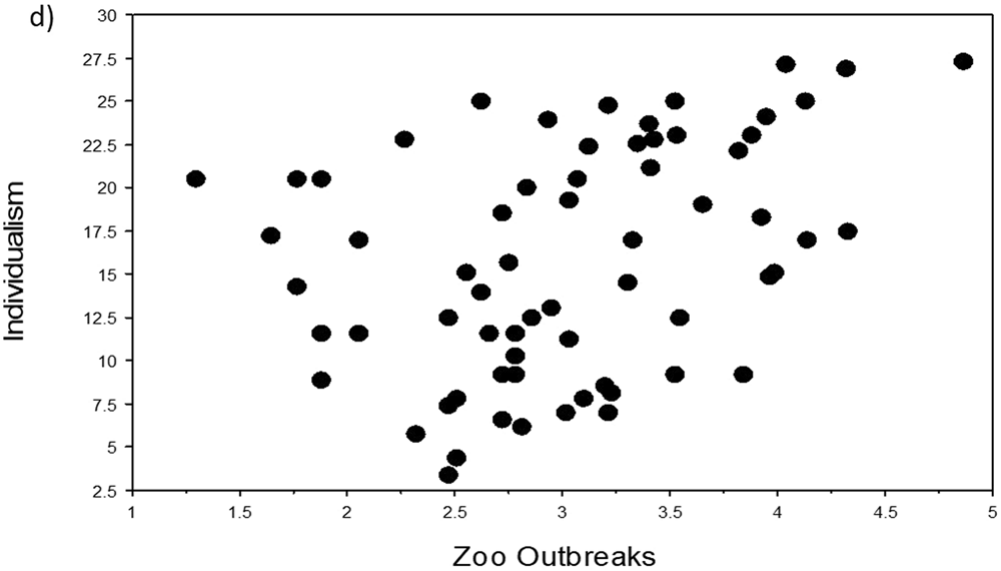
Relationships of Individualism to Zoo Outbreaks (number of zoonotic disease outbreaks). Scatterplot of Individualism (values of collectivism-individualism for n = 66 countries) against the independent variable Zoo Outbreaks (variables were Box-Cox transformed, raw data in Supplementary Table S1).

**Figure 5:**
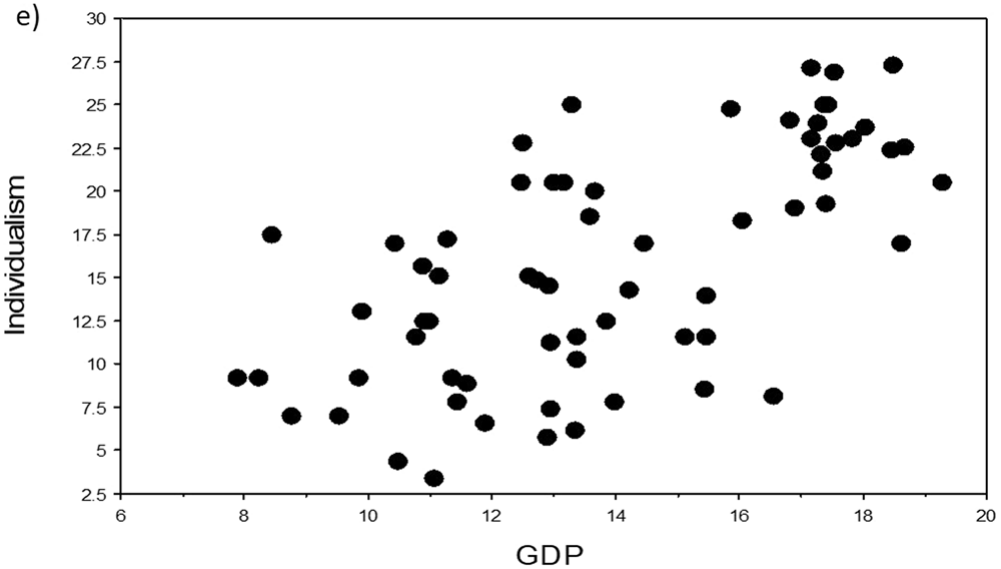
Relationships of Individualism to GDP (GDP per capita). Scatterplot of Individualism (values of collectivism-individualism for n = 66 countries) against the independent variable GDP (variables were Box-Cox transformed, raw data in Supplementary Table S1).

Four variables (namely Area, Surveys, Population, EID Events) scored the lowest VI scores in the two machine learning analyses (columns 2 and 3 in Table 1) and were also all associated with non-significant p-values (with one exception due to outliers) and the lowest rho-values and r^2^-values in the three regression analyses (columns 4–6 in Table 1). The five remaining independent variables all correlated significantly with Individualism in our three regression analyses. In particular, Infect Outbreaks and Zoo Outbreaks were positively and significantly correlated with Individualism, in support of our novel hypothesis that individualistic countries should have been subjected to a higher number of disease outbreaks (Table 1, Figs 1–5).

**Table 1:** Variable importance (VI) scores and regression statistics of nine independent variables tested against the dependent variable Individualism (values of collectivism-individualism for n = 66 countries) ranked according to their VI scores. In machine learning, the most important variable is always given a 100% importance score.

Independent variable	VI untransformed	VI Box-Cox transformed	Spearman-rank	Regression untransformed	Regression Box-Cox transformed
Hist Path	100.0	100.0	*−0.66*, < *0.0001*	*−0.69, 0.47*, < *0.0001*	*−0.68, 0.47*, <*0.0001*
GDP	74.9	73.0	0.63, < 0.0001	0.66, 0.43, < 0.0001	0.65, 0.42, <0.0001
Infect Outbreaks	52.2	55.0	0.47, 0.0002	0.50, 0.25, < 0.0001	0.41, 0.17, 0.0007
Rich Path	49.6	53.9	*−0.54*, < *0.0001*	*−0.45, 0.20, 0.0002*	*−0.48, 0.23, <0.0001*
Zoo Outbreaks	26.1	27.3	0.40, 0.001	0.43, 0.18, 0.0004	0.37, 0.14, 0.002
Area	23.5	23.7	*−0.02, 0.87*	0.13, 0.02, 0.31	0.02, 0.0002, 0.90
Surveys	21.6	23.3	0.15, 0.23	0.14, 0.02, 0.26	0.13, 0.02, 0.31
Population	19.6	22.2	*−0.11, 0.36*	*−0.09, 0.008, 0.47*	*−0.11, 0.01, 0.37*
EID Events	14.7	15.8	*−0.11, 0.36*	0.34, 0.12, 0.005^*^	0.21, 0.04, 0.09^$^

For the Spearman rank correlation, we report the rho-value and p-value. For the linear regression, we report the standard coefficient, the r^2^-value and the p-value; ‘untransformed’ refers to the raw data being used, while ‘Box-Cox transformed’ refers to all variables having been Box-Cox transformed. Negative correlations are printed in italic letters.

^*^This significant p-value is due to the three highest values for EID Events, which are from Australia, the United Kingdom and the United States of America, and which are clear outliers; without them, the values in the respective cell are: 0.15, 0.02, 0.23. ^$^Therefore, even when these three outliers are included, the significant association disappears in the analysis with Box-Cox transformed variables.

### Checking for highly correlated independent variables

We then looked at all the correlations between the five remaining independent variables (Supplementary Table S2) and found only two variables (namely, Infect Outbreaks and Zoo Outbreaks) with a rho-value or r^2^-value > 0.90, while all other correlations had values ≤ 0.65. Given that Infect Outbreaks and Zoo Outbreaks were therefore almost identical variables, we carried out all the following multiple regression analyses with them separately.

### Determining the best final models with several independent variables

Therefore, our first multiple regression analysis included only the independent variables Hist Path, Rich Path, Infect Outbreaks, and GDP. Only two models out of the possible 15 models (using all possible combinations of the four independent variables) had substantial support (Table 2). In the best model, Hist Path and Rich Path were negatively and significantly correlated with Individualism explaining 52% of the variation, and Infect Outbreaks was positively and significantly correlated with Individualism explaining a further 12% of the variation. The second best model excluded the independent variable Rich Path.

**Table 2:** Multiple linear regression models of four independent variables tested against the dependent variable Individualism (values of collectivism-individualism for n = 66 countries) using only Box-Cox transformed variables. For each model, the sample size (n), the F-value, the total P-value of the entire model and its small sample corrected AIC (AIC_*c*_), AIC_*c*_ differences (Δ_*i*_), and Akaike weight (w_*i*_) are given. Only models with substantial support (Δ_*i*_ < 2) are presented (see Methods). For each independent variable within the respective model, the standard coefficient and P-value is given; behind the standard coefficient, the percentage variation explained by the independent variable is given in brackets whereby independent variables which explained most of the unexplained variation were entered first. Negatively correlating independent variables are printed in italic letters.

Dep. variable	n	F	Ind. variable 1		Ind. variable 2		Ind. variable 3		Ind. variable 4		Total P	AIC_*c*_	Δ_*i*_	w_*i*_
Models including Infect Outbreaks			Hist Path		Rich Path		Infect Outbreaks		GDP					
Individualism	66	36.19	*−0.42 (47%)*	*0.0002*	*−0.34* (*5%*)	*0.004*	0.46 (12%)	<0.0001	—	—	<0.0001	89.9	0	0.410
Individualism	66	44.07	*−0.65 (47%)*	<*0.0001*	—	—	0.35 (12%)	<0.0001	—	—	<0.0001	91.5	1.58	0.186
**Models including Zoo Outbreaks**			**Hist Path**		**Rich Path**		**Zoo Outbreaks**		**GDP**					
Individualism	66	36.66	*−0.42 (47%)*	*0.0003*	*−0.38 (6%)*	*0.0016*	0.48 (11%)	<0.0001	—	—	<0.0001	89.7	0	0.385
Individualism	66	28.03	*−0.38 (47%)*	*0.001*	*−0.32 (6%)*	*0.0145*	0.42 (11%)	0.0001	0.14 (1%)	0.24	<0.0001	91.4	1.77	0.159

Our second multiple regression analysis included only the independent variables Hist Path, Rich Path, Zoo Outbreaks, and GDP. Again, only two models out of the possible 15 models had substantial support (Table 2). In the best model, Hist Path and Rich Path were negatively and significantly correlated with Individualism explaining 53% of the variation, and Zoo Outbreaks was positively and significantly correlated with Individualism explaining a further 11% of the variation. The second best model included the independent variable GDP, but GDP was not significantly correlated and explained only a further 1% of the variation. Furthermore, the Δ_*i*_ of this model is 1.77 which is therefore close the exclusion threshold of Δ_*i*_ = 2. Therefore, this second model does not offer much additional information.

To visualize the relationship of Individualism with each of the independent variables, we used TreeNet to graph one-variable partial dependence plots. The results for both analyses were very similar (Supplementary Fig. S1). Each relationship was essentially an S-shaped threshold function, going downwards for Hist Path and Rich Path, and going upwards for Infect Outbreaks, Zoo Outbreaks, and GPD. The scatterplots for untransformed variables (not shown) looked similar in shape to those of the TreeNet plots (Supplementary Fig. S1), while the scatterplots for the Box-Cox transformed variables (Figs. 1–5) appear to be more linear because of our data transformations.

### Testing for spatial autocorrelation

Excluding Area, Surveys, and Population (Table 1), we detected almost no significant spatial autocorrelation in four of our remaining variables (Infect Outbreaks, Zoo Outbreaks, EID Events, GDP) (Supplementary Fig. S2). We detected relatively limited significant spatial autocorrelation for Individualism and Rich Path, but found substantial significant spatial autocorrelation for Hist Path.

## Discussion

We confirmed the previous findings of Fincher *et al*.^3^ that collectivist cultures are more likely to be prevalent in countries with a high historical pathogen burden, using first the historical data compiled by Murray and Schaller^10^ which was not part of Fincher *et al*.’s^3^ original analysis. We then further confirmed the relationship with a previously untested index of pathogen richness which we extracted from the GIDEON database. Since this relationship has now been repeatedly tested and confirmed and its implications discussed (see references in Introduction), we refrain from doing so here.

We further tested our novel hypothesis that, in recent times, countries with individualistic values should have been more susceptible to infectious disease outbreaks, zoonotic disease outbreaks, and emerging infectious disease events. In support, we found positive and highly significant correlations which demonstrate that individualistic countries were subjected to a higher number of infectious disease outbreaks and zoonotic disease outbreaks between 1950 and 2008, but we found no support for such a correlation for emerging infectious disease events between 1940 and 2004. These significant correlations explained between 14–25% of the variation of the collectivism-individualism continuum of the 66 tested countries (Table 1). When entered into a multiple regression, the variation explained by the number of infectious disease outbreaks and zoonotic disease outbreaks decreased to 11–12% but nevertheless remained highly significant (Table 2).

The number of infectious disease outbreaks and zoonotic disease outbreaks also correlate positively with four variables which could be argued to positively influence their magnitudes, especially the number of surveys carried out, but also the area, GDP, and human population size of each country (Supplementary Table S2). However, we found that none of these potentially confounding variables had any influence in a multiple regression, whether we looked at additional variation explained, P-values, or Δ_*i*_ values.

Despite the relatively small amount of variation explained, we should therefore consider the possibility that the proposed protective function of collectivist values, lifestyles and behaviours may also provide a protective (although somewhat limited) function against infectious disease outbreaks and zoonotic disease outbreaks which continues into the present. Or, reversely, one possible cost of individualistic countries may be their higher susceptibility towards disease outbreaks. If we accept such a causal relationship, then the shapes of the relationships are also of interest as they are all S-shaped threshold functions (Supplementary Fig. S1). Therefore, instead of being a linear relationship whereby a certain increase in collectivist behaviours leads to a proportional decrease in disease outbreaks, it may rather be that a certain threshold of collectivist behaviours has to be reached before the protective function of these behaviours ‘kicks in.’

Given the correlational nature of our evidence, we also need to discuss possible alternative explanations and hypotheses for the positive correlations between individualistic values and the number of outbreaks of infectious diseases and zoonotic diseases. One possible confounding influence is spatial autocorrelation^11^. However, we found no significant spatial autocorrelation for four variables and only limited significant spatial autocorrelation for two further variables. Only for Hist Path did we detect substantial and significant spatial autocorrelation. The significant autocorrelation which we found was at small distances for Individualism, Hist Path and Rich Path and at large distances for Hist Path and Rich Path across countries of similar latitudinal environments. Such spatial autocorrelation for Individualism was previously detected by two studies^7,12^, and the spatial autocorrelation for Hist Path and Rich Path at small and large distances is similar to the already known latitudinal gradient in disease richness and in biodiversity richness^12^. The lack of spatial autocorrelation for Zoo Outbreaks may be due to the increasing connectivity between countries which favours the global spread of infectious diseases^13^. Given the limited amount of spatial autocorrelation which we detected, we suggest that spatial autocorrelation does not invalidate our overall results except perhaps in the case of Hist Path. Therefore, the results involving Hist Path should be considered with caution. Most importantly, however, is that the lack of spatial autocorrelation for Infect Outbreaks, Zoo Outbreaks, and EID Events means that our novel hypothesis tested here for the first time is not affected by spatial autocorrelation.

Another alternative explanation for the statistical association between individualism and the number of infectious disease outbreaks and zoonotic disease outbreaks is that collectivist values and behaviours are generated for other reasons, e.g., religious beliefs, the need for defence against neighbours, or the perpetuation by charismatic leaders. The decreased spread of diseases would thus be only a beneficial side effect of these collectivist values and behaviours.

Another possibility is that individualistic countries tend to be richer (Table 1), and therefore people are able to afford more travel, which in turn should increase contact rates, which in turn could increase the number of disease outbreaks. Studies of mobility^14^ combined with modelling studies^15^ could be helpful to investigate this explanation. However, even if there was no causal link between collectivist values and disease outbreaks at all, the mere fact that, for whatever reason, individualistic countries face a somewhat elevated threat of disease outbreaks is an important piece of scientific and public health information.

In a series of insightful papers, Hruschka and his colleagues^16–18^ have proposed further alternative hypotheses or possibly confounding variables, namely the life history theory (pertaining to an individual’s life history strategy), the material or existential security hypothesis (pertaining to the absence or existence of effective large-scale social institutions and public health infrastructure which provide individuals with a safety net), and differences in ethnic groups and religious affiliations. Therefore, our results are limited in the sense that we did not test for these possibly confounding variables. However, since some of these variables (e.g., education and public health) are strongly correlated with GDP, we indirectly tested for them. Nevertheless, including additional independent variables of education, public health, religion, etc., would indeed be a further improvement of our analysis. These authors also advocate subdividing the analysis into continents or regions (e.g., the West versus the Rest). However, for the initial test of our novel hypothesis, we wanted to provide a global analysis, which naturally cannot be subdivided.

These and other authors have also raised concerns about ‘observational’ studies (in contrast to ‘experimental’ studies) which interpret correlations between parasite variables and other cultural variables at the country-level or state-level. Here, they mostly refer to the possible threat of pseudoreplication because of similar cultural descent. However, we here side strongly with Thornhill and Fincher’s^19^ reasoning that different nations are statistically independent sampling units and refer the reader to their long and detailed discussion of this important epistemological and logical issue which goes to the heart of the scientific method.

Against our expectation, there was no correlative evidence for the number of emerging infectious disease events between 1940 and 2004. We therefore suggest that the reasons for a new disease to emerge are rather different from the reasons for the outbreaks of more ‘established’ infectious diseases and zoonotic diseases. Jones *et al*.^9^ related EID events to human population density and growth, latitude, and wildlife host richness. Just like the countries’ area and human population size are not related to individualism (Table 1), human population density is not related to individualism either (unpublished results). However, we did not test for latitude or wildlife host richness in this study. Further studies with more independent variables are therefore called for to distinguish the variables which may explain the possible reasons for disease outbreaks versus disease emergence.

Finally, we propose that there is considerable merit in combining more traditional statistical approaches (null hypothesis significance testing using regression models and model comparisons using AIC values) with newly developed and more advanced statistical analyses (TreeNet machine learning algorithms) because (1) they illuminate different aspects of the ‘signal’ in the data and (2) and they give greater confidence in the results when they agree, as in our case.

Pathogen burden (historical or contemporary) is positively correlated with repression of individual rights and negatively correlated with social and political liberalism^8,10^, and collectivist societies are often characterized by a high level of xenophobic attitudes^20^ and anti-science attitudes^7^. While our findings should in no way be interpreted to support such moral and political attitudes, future research should investigate what the exact protective behaviours and mechanisms of collectivist societies are which could inhibit infectious disease outbreaks and zoonotic disease outbreaks, and, more generally, the spread and transmission of pathogens and parasites. Since we live in a more and more interconnected world where pathogens and parasites can easily travel with goods and people, the risks that infectious diseases will spread may be enhanced by the individualistic values which are increasing worldwide^21^ and which promote free trade, free movement of people, and xenophilic attitudes. We should therefore learn what countermeasures we can take, which may include the protective behaviours but not necessarily the xenophobic attitudes of collectivist societies. Such protective behaviours and mechanisms could then become part of preparedness plans which deal with the threats of disease outbreaks, emerging diseases, and even bioterrorism (e.g., ref.^22^).

We finally advocate that future studies seek to broadly integrate separate findings which concern the origins and distributions of infectious diseases and zoonotic diseases^12,23,24^, the socio-ecological variables explaining disease emergence and epidemics^9^, and their links with the variation in cultural values^7,25^. Such comprehensive studies should increase our understanding of the distribution and potential spread of diseases, and how we can be prepared in order to contain them as best as possible, including the behaviour from individual people up to entire countries and the global human society.

## Methods

### Dependent variable Individualism

Fincher *et al*.^3^ used four measures of the collectivism-individualism continuum which they collated from four different sources. They showed that these four measures were highly correlated amongst themselves, and that they were also all correlated with their measures of pathogen burden across countries. Therefore, we used only one of these four datasets, namely the one originally collated by Hofstede^2^. Hofstede’s^2^ dataset rendered collectivism-individualism scores for 66 countries (Supplementary Table S1) with lower scores indicating greater collectivism, and higher scores indicating greater individualism.

### Independent variables

We used two measures to quantify each country’s historical pathogen burden (abbreviated Hist Path and Rich Path) and three measures to quantify each country’s recent number of infectious disease outbreaks, zoonotic disease outbreaks, and emerging infectious disease events (abbreviated Infect Outbreaks, Zoo Outbreaks, and EID Events):

1. Hist Path: We used the measure of historical pathogen burden developed by Murray and Schaller^10^ which we mentioned in the Introduction.
2. Rich Path: We used the total number (or richness) of pathogens and parasites as a measure of endemic pathogen burden. To collate the total number of pathogens and parasites, Morand *et al*.^26^ extracted the relevant data from GIDEON (Global Infectious Disease and Epidemiology Online Network, https://www.gideononline.com/) which contains information on the presence and occurrence of epidemics of human infectious diseases in each country as well as the number of surveys conducted in each country. This dataset is generally considered to be the most complete and up-to-date in the world and has been regularly used in previous comparative studies of pathogen diversity and epidemics (e.g., refs^12,24,26,27^). Rich Path is similar but not the same as Hist Path (the r^2^ of these two measures is only 0.41, see Supplementary Table S2).
3. Infect Outbreaks: We also used GIDEON to collate the total number of infectious disease outbreaks over the years 1950–2008 using methods outlined in Morand *et al*.^26^. The definition of “infectious disease outbreak” is given in Supplementary Text S1 online.
4. Zoo Outbreaks: We also used GIDEON to collate the total number of zoonotic disease outbreaks over the years 1950–2008 using methods outlined in Morand *et al*.^26^. The definition of “zoonotic disease outbreak” is given in Supplementary Text S1.
5. EID Events: We used the total number of emerging infectious disease events between 1940 and 2004 compiled by Jones *et al*.^9^. The definition of “emerging infectious disease events” is given in Supplementary Text S1.
  For each country, we also compiled data for four other variables which may reasonably be assumed to influence (or correlate with) the collectivism-individualism continuum as well as the five pathogen measures described above:
6. Surveys: We also used GIDEON to collate the total number of surveys conducted in each country as a variable which measures sampling effort because sampling effort is known to positively influence the recorded number of pathogens (Hist Path and Rich Path) as well as the recorded number of outbreaks (Infect Outbreaks and Zoo Outbreaks) (cf. Walther *et al*.^28^ for effects of sampling effort).
7. Area: We used the data from the World Bank given in km^2^.
8. GDP per capita: We used the data for the year 2000 from the World Bank given in US dollars.
9. Population: We used the data for the year 2000 from the World Bank given in the number of human individuals.

All raw data used for our analyses are available in Supplementary Table S1.

### Statistical analyses

For the purpose of our analysis, we agree with Thornhill and Fincher’s^19^ reasoning that different nations are statistically independent sampling units because our investigated question is one of the persistence (and not the origin) of cultural traits.

We attempted to bring together two rather different statistical methods in order to better explore the data. We compare our approach to using two different kinds of microscopes, each which can tell us different attributes of our data.

1. Machine learning: We used machine learning to rank the importance of each independent variable and to be able to display non-linear relationships between the independent and dependent variables. The latest version of the stochastic gradient boosting classification and regression tree algorithm TreeNet as implemented in the Salford Systems Data Mining and Predictive Analytics Software (https://www.salford-systems.com/) was used. Through the use of a non-linear and non-parametric machine learning algorithm such as TreeNet, several of the traditional statistical problems, such as to extract ‘a signal’ from complex data and to generalize these patterns from the data (*sensu* refs^29,30^), can be overcome. Furthermore, non-linear relationships can be graphically displayed.

The TreeNet algorithm is based on a regression tree analysis in which the algorithm recursively partitions the entire dataset into two partial datasets based on the independent variables. This is achieved by using an optimized set of independent variables in order to create binary trees which try to minimize variation within each dataset, whereby subsequent trees are constructed for the prediction of the residuals from the previous trees, and results are then computed from the entire group of trees^31^. Specifically, the stochastic gradient boosting method was used which further optimizes performance by maintaining a running tally which avoids overfitting (similar to bagging^31^). The pre-set default settings for TreeNet were used which are known to generally perform very well in most cases (e.g., ref.^32^; F. Huettmann, personal communication). The maximum number of trees to be used was set to 10000, and the learning rate was set to 0.0005. The maximum number of nodes per tree was set between six and ten^33^. We applied 3–10 minimum samples for terminal branches and a 10-fold cross-validation. In our case, we used TreeNet because (1) it assigns a variable importance (VI) score to each independent variable which allows one to rank variables by importance, and (2) it generates one-variable partial dependence plots which graphically display the relationship between one independent variable and the dependent variable. However, a partial dependence plot does not show the raw relationship between the independent and the dependent variable. Instead, the plotted function depicts how the value of the independent variable influences the model predictions (or dependent variable) after the influence of all other independent variables has been “averaged out.” The main advantage of these partial dependence plots is that they can be constructed for any predictive model, regardless of its form or its complexity. Therefore, partial dependence plots do not ignore the effect of all the other predictors; rather, they average out the effects of the other predictors from the full model. Consequently, the resulting plot can look quite different to the scatterplot of the independent versus the dependent variable.

2. Significance testing and model comparisons: We used null hypothesis significance testing and model comparison using AIC values within the framework described by Burnham and Anderson^34^. We performed multiple linear regression with SPSS version 19 to correlate the dependent variable Individualism with the independent variables. We ranked the support for competing models using the likelihood-based methods based on the information theoretic approach proposed in Burnham and Anderson^34^. We ranked models based on the Akaike’s Information Criterion adjusted for small sample size (AIC_*c*_), and we accepted only those models with an AIC_*c*_ difference (Δ_*i*_) < 2 as having ‘substantial’ support (*sensu* page 70 in reference ^34^; see also refs^35–37^). These are also the models with the largest Akaike weights (w_*i*_).

Several of our variables (Rich Path, Infect Outbreaks, Zoo Outbreaks, EID Events, Area, GDP, Population) were very right-skewed. Therefore, we Box-Cox-transformed^38^ all of our variables to achieve a normal distribution (tested by the one-sample Kolmogorov-Smirnov test and the Shapiro-Wilk test, all variables had P > 0.05 after their transformation). To further test for the definite existence of a relationship between two variables, we also used the Spearman-rank correlation as applied in SPSS version 19. The alpha level for significance was P < 0.05 for all of our tests, and all tests were two-tailed.

We investigated the level of spatial autocorrelation in our variables with the package spatialEco^39^ implemented in the freeware R. We used this package to generate correlograms for independent and dependent variables. The correlograms depict spatial autocorrelation as a function of distance. If there is no spatial autocorrelation, the values will be near zero and within the blue area which signifies non-significance. Values outside the blue area signify significant spatial autocorrelation, whereby (1) positive values indicate that high attribute values cluster near other high values, or low attribute values cluster near other low values while (2) negative values indicate that high attribute values repel other high values, and tend to be near low values.

## Electronic supplementary material

Supplementary information
