# Supplementary material for: Individualistic values are related to an increase in the outbreaks of infectious diseases and zoonotic diseases
Source: Sci Rep. 2018 Mar 1;8:3866. doi: 10.1038/s41598-018-22014-4 (PMC5832805; doi:10.1038/s41598-018-22014-4)

Supplementary Information (one Text, two Tables, and two Figures) for:

**Individualistic values are related to an increase in the outbreaks of infectious diseases and zoonotic diseases**

Serge Morand^1,2,3^ & Bruno A. Walther^4*^

1 November 2017

^1^ *CIRAD, UMR ASTRE, F-34398, Montpellier, France*

^2^ *CNRS – Institut des Sciences de l’Evolution de Montpellier, Université de Montpellier, France*

^3^ *Faculty of Veterinary Technology, Kasetsart University, Bangkok, Thailand*

^4^*Master Program in Global Health and Development, College of Public Health, Taipei Medical University, 250 Wu-Hsing St., Taipei 110, Taiwan*

**Text S1.** Detailed definitions of infectious disease outbreaks (Infect Outbreaks), zoonotic disease outbreaks (Zoo Outbreaks), and emerging infectious disease events (EID Events).

**Infectious disease outbreaks** (Infect Outbreaks):

The WHO (2017a) defined a disease outbreak as follows: “A disease outbreak is the occurrence of cases of disease in excess of what would normally be expected in a defined community, geographical area or season. An outbreak may occur in a restricted geographical area, or may extend over several countries. It may last for a few days or weeks, or for several years.”

This definition is also used for entry into the GIDEON database, and therefore we follow their definition. Furthermore, GIDEON Informatics (2017) under the question “How are outbreaks defined in GIDEON?” states that the designation “outbreak” may appear for one of four reasons:

1. An event is specifically [sic] reported as “an outbreak” in source literature.

2. In general, any grouping of cases – including family clusters and epidemics – will be listed as an “outbreak” for purpose of consistency. The term “outbreak” is generic here, and much will depend on the nature of the disease itself as there is no numerical cutoff.

3. Citations of animal disease are denoted as “outbreaks” – even when only one animal is involved – in keeping with OIE [i.e., World Organisation for Animal Health] definitions. Thus, a report of anthrax in a single goat is considered an outbreak in their reporting system.

**Zoonotic disease outbreaks** (Zoo Outbreaks):

The WHO (2017b) defined a zoonosis as follows: “A zoonosis is any disease or infection that is naturally transmissible from vertebrate animals to humans. Animals thus play an essential role in maintaining zoonotic infections in nature. Zoonoses may be bacterial, viral, or parasitic, or may involve unconventional agents. As well as being a public health problem, many of the major zoonotic diseases prevent the efficient production of food of animal origin and create obstacles to international trade in animal products.”

This definition is also used for entry into the GIDEON database, and therefore we follow this definition.

**Emerging infectious disease events** (EID Events):

Jones et al. (2017) defined emerging infectious disease events as follows: “Here we define the first temporal origination of an EID (that is, the original case or cluster of cases representing an infectious disease emerging in human populations for the first time …) as an EID ‘event’.” Since we extracted all our data from Jones et al. (2017), we follow this definition.

We are aware that different authors and researchers may use different definitions of infectious diseases, zoonotic diseases, and emerging infectious diseases than the ones used in GIDEON and presented above. However, a discussion about the pros and cons of different definitions is beyond the scope of our study.

References:

GIDEON Informatics. Global Infectious Disease and Epidemiology Online Network (2017). https://www.gideononline.com/faq/

Jones, K. E. *et al*., Global trends in emerging infectious diseases. *Nature* **451,** 990–993 (2008).

WHO. Disease outbreaks (2017a). http://www.who.int/topics/disease_outbreaks/en/

WHO. Zoonoses (2017b). http://www.who.int/topics/zoonoses/en/

**Table S1.** Data for 66 countries used in our analysis. For column names and definitions, see Methods in main text.

| **Country** | **Individualism** | **Hist Path** | **Rich Path** | **Infect Outbreaks** | **Zoo Outbreaks** | **EID Events** | **Surveys** | **Area** | **GDP** | **Population** |
| --- | --- | --- | --- | --- | --- | --- | --- | --- | --- | --- |
| Argentina | 46 | -0.12 | 220 | 36 | 57 | 3 | 73 | 2775401 | 7694 | 36938728 |
| Australia | 90 | -0.25 | 211 | 64 | 176 | 17 | 82 | 7662592 | 21768 | 19153000 |
| Austria | 55 | -0.77 | 194 | 24 | 37 | 1 | 57 | 84906 | 23866 | 8011561 |
| Bangladesh | 20 | 0.62 | 207 | 12 | 24 | 2 | 42 | 135887 | 335 | 140766909 |
| Belgium | 75 | -1.00 | 194 | 29 | 32 | 0 | 50 | 31318 | 22666 | 10252000 |
| Bosnia -Herzegovina | 27 | 0.00 | 200 | 14 | 22 | 2 | 14 | 51385 | 1491 | 3693578 |
| Brazil | 38 | 0.93 | 251 | 62 | 156 | 6 | 104 | 8420640 | 3701 | 174174447 |
| Bulgaria | 30 | -0.35 | 204 | 16 | 17 | 1 | 21 | 119439 | 1601 | 8060000 |
| Canada | 80 | -1.31 | 214 | 73 | 206 | 8 | 66 | 9590309 | 23560 | 30769700 |
| Chile | 23 | -0.45 | 204 | 25 | 26 | 0 | 61 | 668869 | 4878 | 15418704 |
| China | 20 | 1.03 | 246 | 61 | 127 | 7 | 108 | 9344594 | 949 | 1262645000 |
| Colombia | 13 | 0.27 | 236 | 16 | 24 | 0 | 57 | 1153540 | 2523 | 39772905 |
| Costa Rica | 15 | 0.12 | 215 | 17 | 17 | 1 | 23 | 50525 | 4057 | 3930863 |
| Croatia | 27 | -0.44 | 204 | 21 | 26 | 4 | 32 | 53733 | 4856 | 4426000 |
| Czech Republic | 58 | -0.87 | 205 | 25 | 28 | 3 | 41 | 80165 | 5521 | 10273300 |
| Denmark | 74 | -0.98 | 191 | 40 | 64 | 2 | 43 | 32408 | 29993 | 5337344 |
| Ecuador | 8 | 0.34 | 222 | 15 | 18 | 1 | 34 | 248706 | 1295 | 12310314 |
| El Salvador | 19 | 0.30 | 205 | 7 | 8 | 0 | 21 | 20886 | 2209 | 5945317 |
| Estonia | 60 | -0.62 | 200 | 5 | 7 | 0 | 14 | 38745 | 4144 | 1369513 |
| Finland | 63 | -0.75 | 194 | 38 | 65 | 1 | 46 | 333518 | 23514 | 5176198 |
| France | 71 | -0.46 | 214 | 69 | 136 | 8 | 74 | 550788 | 21914 | 58895517 |
| Germany | 67 | -0.87 | 203 | 52 | 122 | 10 | 80 | 349898 | 23114 | 82210000 |
| Greece | 35 | 0.08 | 204 | 23 | 21 | 1 | 62 | 105140 | 11501 | 10917500 |
| Guatemala | 6 | 0.42 | 214 | 13 | 17 | 0 | 29 | 108418 | 1718 | 11230704 |
| Hungary | 80 | -1.00 | 200 | 26 | 21 | 1 | 35 | 95569 | 4690 | 10210971 |
| India | 48 | 0.94 | 246 | 60 | 288 | 6 | 95 | 3151251 | 453 | 1015923000 |
| Indonesia | 14 | 0.63 | 238 | 17 | 48 | 3 | 56 | 1697375 | 804 | 205280270 |
| Iran | 41 | -0.15 | 217 | 17 | 25 | 0 | 75 | 1614726 | 1584 | 63938646 |
| Ireland | 70 | -0.45 | 189 | 32 | 66 | 0 | 34 | 67817 | 25380 | 3805400 |
| Israel | 54 | 0.52 | 201 | 55 | 94 | 3 | 70 | 31135 | 19836 | 6289000 |
| Italy | 76 | 0.16 | 210 | 58 | 151 | 7 | 84 | 296127 | 19269 | 56948600 |
| Jamaica | 39 | 0.18 | 194 | 19 | 19 | 0 | 28 | 9037 | 3479 | 2589389 |
| Japan | 46 | 0.43 | 215 | 71 | 208 | 13 | 75 | 358359 | 36789 | 126870000 |
| Latvia | 70 | -0.62 | 198 | 9 | 13 | 0 | 23 | 62353 | 3302 | 2372000 |
| Lithuania | 60 | -0.75 | 197 | 7 | 8 | 0 | 18 | 64802 | 3267 | 3499527 |
| Luxembourg | 60 | -1.11 | 187 | 4 | 4 | 0 | 8 | 2696 | 46458 | 436300 |
| Malaysia | 26 | 0.50 | 231 | 25 | 37 | 5 | 59 | 323151 | 4030 | 23273615 |
| Mexico | 30 | 0.28 | 233 | 43 | 80 | 0 | 68 | 1923122 | 5935 | 97966000 |
| Morocco | 46 | 0.59 | 203 | 9 | 10 | 0 | 41 | 411474 | 1270 | 28827115 |
| Netherlands | 80 | -0.87 | 196 | 55 | 77 | 3 | 60 | 32983 | 24180 | 15925431 |
| New Zealand | 79 | -0.98 | 189 | 29 | 48 | 0 | 36 | 249004 | 13336 | 3857800 |
| Norway | 69 | -0.85 | 194 | 36 | 59 | 1 | 34 | 349505 | 37472 | 4491000 |
| Pakistan | 14 | 0.02 | 217 | 22 | 36 | 2 | 40 | 871274 | 536 | 138080000 |
| Panama | 11 | 0.09 | 220 | 14 | 14 | 0 | 24 | 73322 | 3938 | 2950801 |
| Peru | 16 | 0.23 | 227 | 24 | 41 | 2 | 68 | 1302897 | 2049 | 26004162 |
| Philippines | 32 | 0.50 | 222 | 18 | 33 | 1 | 45 | 232173 | 977 | 77689369 |
| Poland | 60 | -0.87 | 199 | 26 | 39 | 1 | 51 | 305676 | 4454 | 38453757 |
| Portugal | 27 | 0.47 | 203 | 15 | 10 | 0 | 43 | 93103 | 11443 | 10225803 |
| Republic of Korea | 18 | -0.11 | 217 | 31 | 47 | 1 | 82 | 91306 | 11347 | 47008000 |
| Romania | 30 | -0.18 | 202 | 21 | 29 | 0 | 33 | 231660 | 1651 | 22443000 |
| Russian Federation | 39 | -0.39 | 235 | 52 | 162 | 3 | 48 | 16600000 | 1775 | 146303000 |
| Slovakia | 52 | -1.00 | 201 | 16 | 24 | 0 | 22 | 47844 | 5326 | 5388741 |
| Slovenia | 27 | -0.87 | 204 | 11 | 8 | 2 | 24 | 20441 | 9999 | 1989000 |
| Spain | 51 | -0.05 | 205 | 58 | 145 | 4 | 93 | 485473 | 14422 | 40263200 |
| Suriname | 47 | 0.63 | 215 | 8 | 6 | 0 | 10 | 149500 | 1910 | 467162 |
| Sweden | 71 | -0.98 | 194 | 43 | 78 | 5 | 52 | 433619 | 27879 | 8869000 |
| Switzerland | 68 | -1.08 | 196 | 28 | 42 | 5 | 54 | 41452 | 34787 | 7184222 |
| Taiwan | 17 | 0.30 | 212 | 34 | 49 | 5 | 50 | 32920 | 17400 | 22191090 |
| Thailand | 20 | 0.64 | 235 | 44 | 77 | 7 | 89 | 506001 | 1968 | 62346822 |
| Trinidad & Tobago | 16 | -0.03 | 203 | 15 | 18 | 3 | 32 | 4857 | 6296 | 1295100 |
| Turkey | 37 | 0.16 | 214 | 43 | 55 | 1 | 90 | 790419 | 4011 | 66459578 |
| United Kingdom | 89 | -1.01 | 200 | 77 | 286 | 27 | 61 | 219141 | 25089 | 58892514 |
| United States of Am. | 91 | -0.89 | 240 | 135 | 788 | 105 | 113 | 9400722 | 35081 | 282172000 |
| Uruguay | 36 | 0.39 | 204 | 9 | 7 | 0 | 20 | 175471 | 6914 | 3300847 |
| Venezuela | 12 | 0.48 | 230 | 15 | 27 | 2 | 53 | 929507 | 4819 | 24311000 |
| Vietnam | 20 | 0.61 | 223 | 20 | 26 | 3 | 59 | 333168 | 402 | 77635400 |

**Table S2.** The upper-right triangle gives the rho-value and p-value for each Spearman rank correlation, and the lower-left triangle gives the r^2^-value and p-value for a simple linear regression (both for Box-Cox transformed variables). Negative correlations are printed in italic letters. The sample size is 66 countries (raw data in Table S1).

| Variable | Hist Path | Rich Path | Infect Outbreaks | Zoo Outbreaks | EID Events | Surveys | Area | GDP | Population |
| --- | --- | --- | --- | --- | --- | --- | --- | --- | --- |
| Hist Path | - | 0.65, p < 0.0001 | *-0.15, p = 0.24* | *-0.07, p = 0.58* | 0.05, p = 0.70 | 0.18, p = 0.14 | 0.27, p = 0.03 | *-0.62, p < 0.0001* | 0.36, p = 0.004 |
| Rich Path | 0.41, p < 0.0001 | - | 0.16, p = 0.19 | 0.23, p = 0.06 | 0.35, p = 0.005 | 0.47, p = 0.0002 | 0.64, p < 0.0001 | *-0.51, p < 0.0001* | 0.67, p < 0.0001 |
| Infect Outbreaks | *0.01, p = 0.43* | 0.08, p < 0.02 | - | 0.96, p < 0.0001 | 0.72, p < 0.0001 | 0.80, p < 0.0001 | 0.49, p < 0.0001 | 0.45, p = 0.0003 | 0.56, p < 0.0001 |
| Zoo Outbreaks | *0.003, p = 0.67* | 0.12, p = 0.004 | 0.93, p < 0.0001 | - | 0.74, p < 0.0001 | 0.80, p < 0.0001 | 0.56, p < 0.0001 | 0.37, p = 0.003 | 0.64, p < 0.0001 |
| EID Events | *0.000, p = 0.99* | 0.13, p = 0.003 | 0.53, p < 0.0001 | 0.56, p < 0.0001 | - | 0.64, p < 0.0001 | 0.38, p = 0.002 | 0.26, p = 0.04 | 0.53, p < 0.0001 |
| Surveys | 0.04, p = 0.09 | 0.28, p < 0.0001 | 0.67, p < 0.0001 | 0.67, p < 0.0001 | 0.40, p < 0.0001 | - | 0.65, p < 0.0001 | 0.18, p = 0.16 | 0.78, p < 0.0001 |
| Area | 0.06, p = 0.04 | 0.46, p < 0.0001 | 0.30, p < 0.0001 | 0.37, p < 0.0001 | 0.15, p = 0.002 | 0.44, p < 0.0001 | - | *-0.21, p = 0.10* | 0.75, p < 0.0001 |
| GDP | *0.38, p < 0.0001* | *0.24, p < 0.0001* | 0.17, p = 0.0005 | 0.10, p = 0.01 | 0.06, p = 0.051 | 0.02, p = 0.24 | *0.05, p = 0.08* | - | *-0.25, p = 0.04* |
| Population | 0.15, p = 0.002 | 0.49, p < 0.0001 | 0.37, p < 0.0001 | 0.48, p < 0.0001 | 0.28, p < 0.0001 | 0.64 , p < 0.0001 | 0.57, p < 0.0001 | *0.08, p = 0.02* | - |

**Figure S1. Dependence plots for independent variables.** One-variable partial dependence plots generated by Treenet for our first analysis of the independent variables Hist Path, Rich Path, Infect Outbreaks, and GDP (A1-A4), and for our second analysis of the independent variables Hist Path, Rich Path, Zoo Outbreaks, and GDP (B1-B4). The sample size is 66 countries (raw data in Table S1).

A1


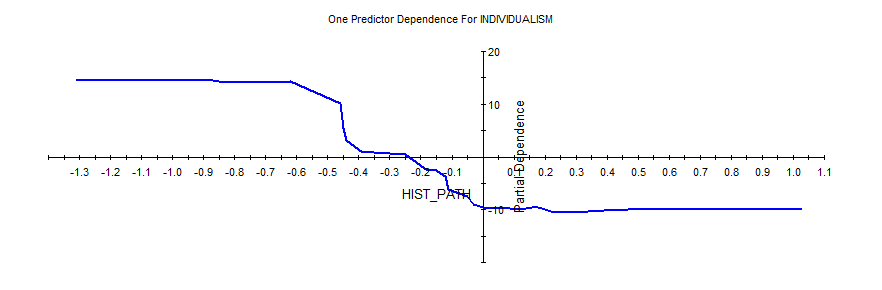


A2


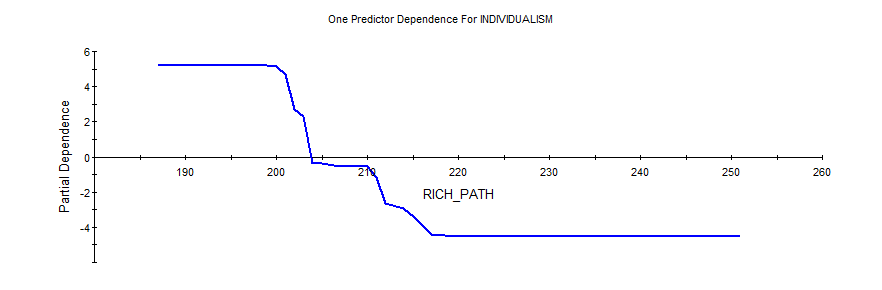


A3


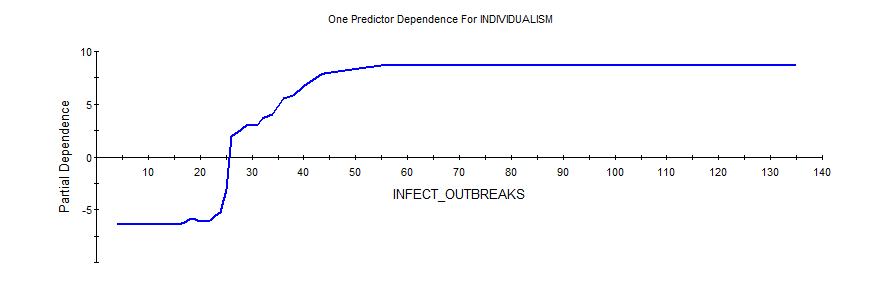


A4


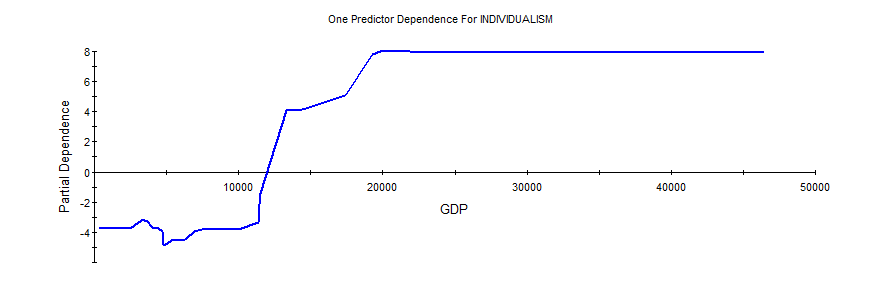


B1


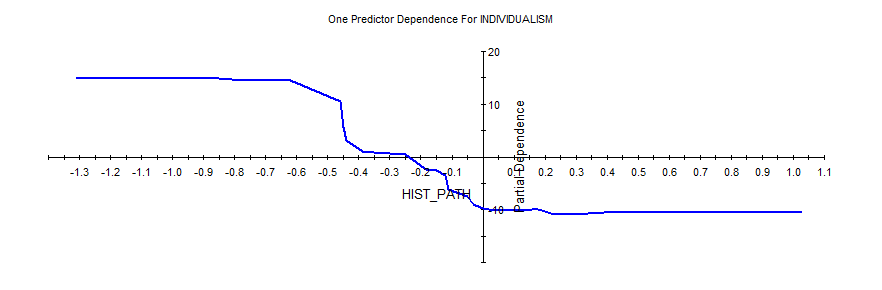


B2


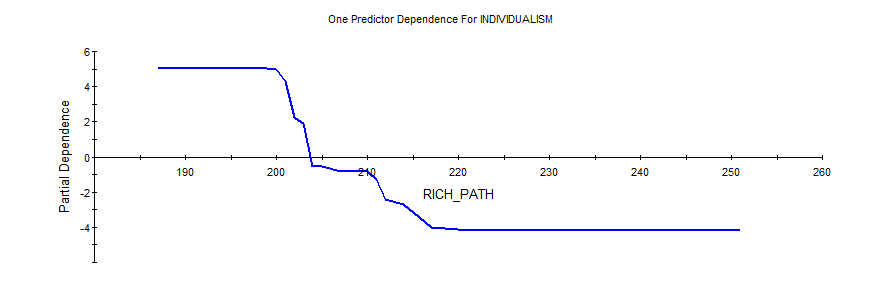


B3


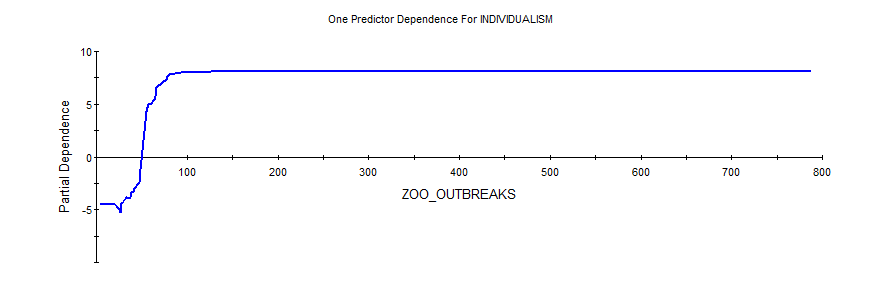


B4


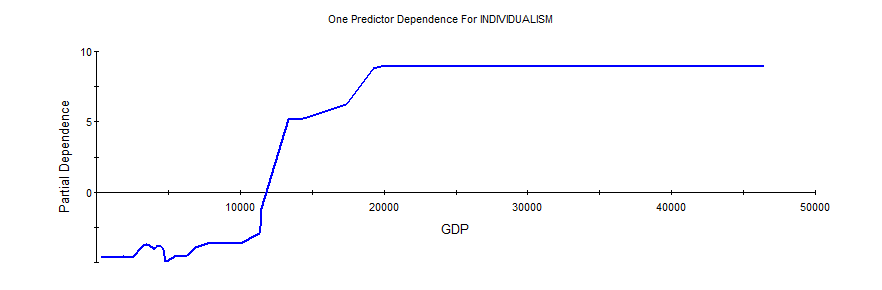


**Figure S2. Test for spatial autocorrelation of variables.** Global maps and spatial autocorrelation correlograms (in km on the x-axis) for the following variables: (a) Individualism; (b) Hist Path; (c) Rich Path; (d) Infect Outbreaks; (e) Zoo Outbreaks; (f) EID Events; (g) GPD. In the correlograms, only points outside the blue zone denote significant spatial autocorrelation. Sample size is 66 countries (Table S1). All maps and correlograms were produced with the freely available package spatialEco (Evans, J. S. 2017. Spatialeco. R package version 0.0.1-7 https://CRAN.R-project.org/package=spatialEco) implemented in the open-access freeware R (https://www.r-project.org/).

(a)


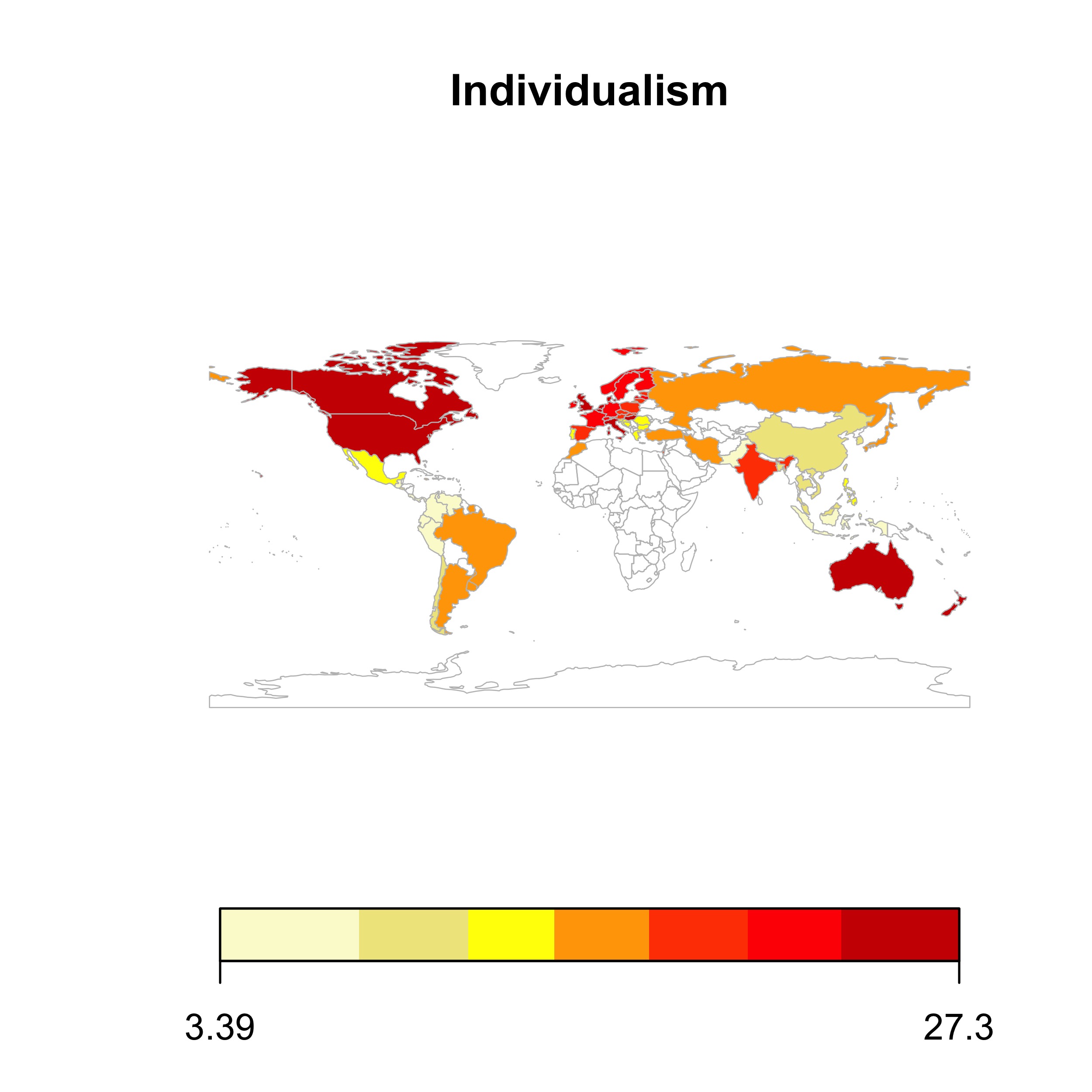


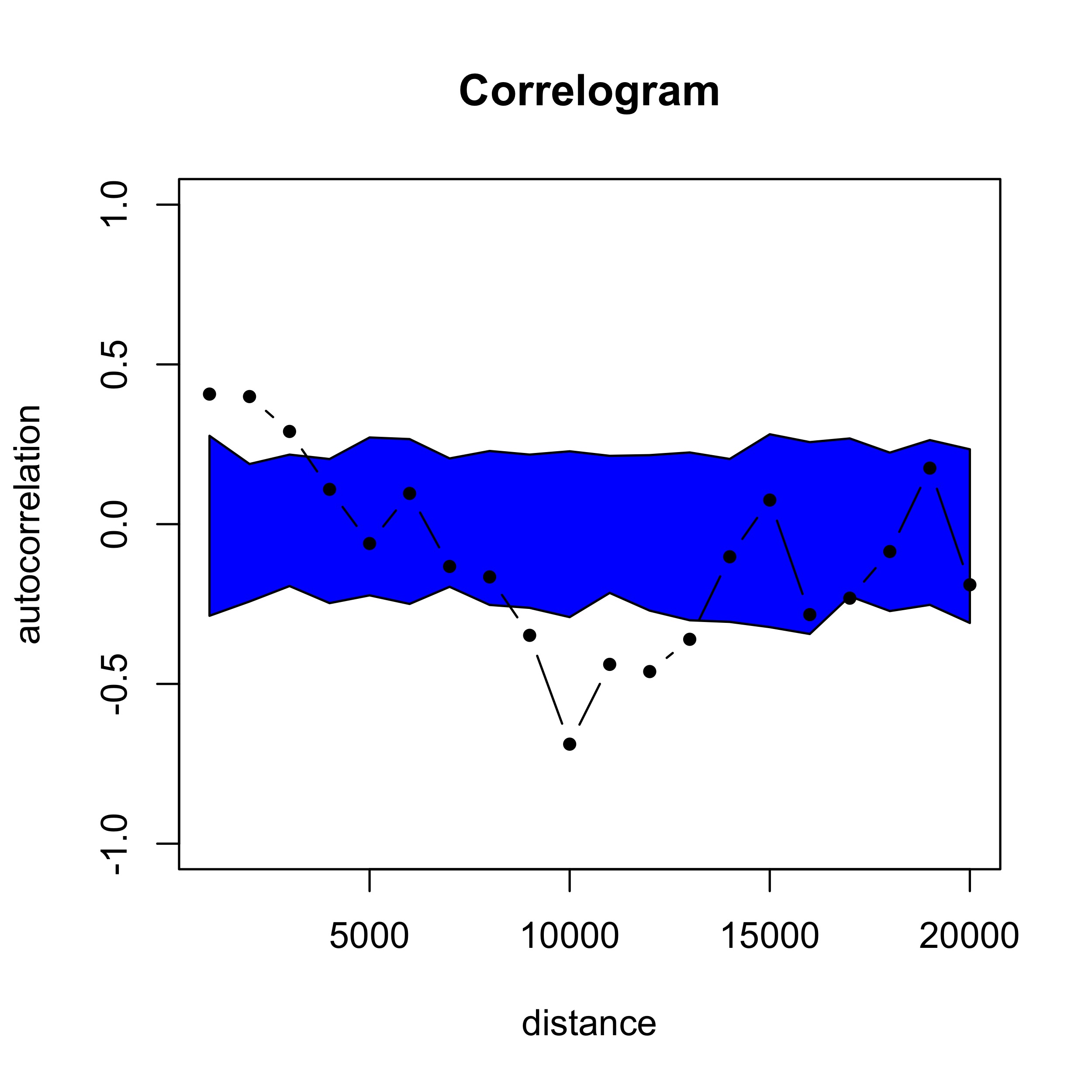


(b)


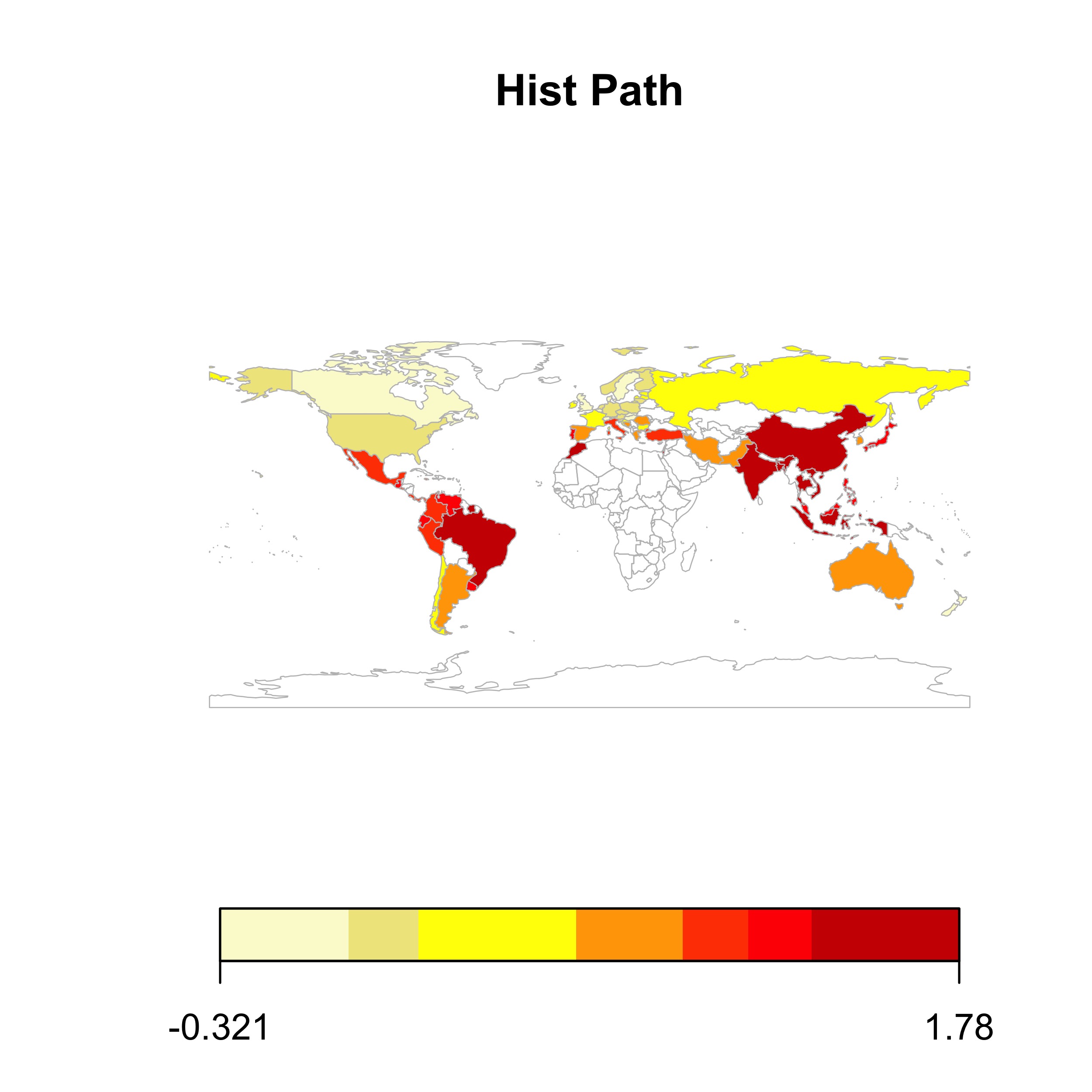


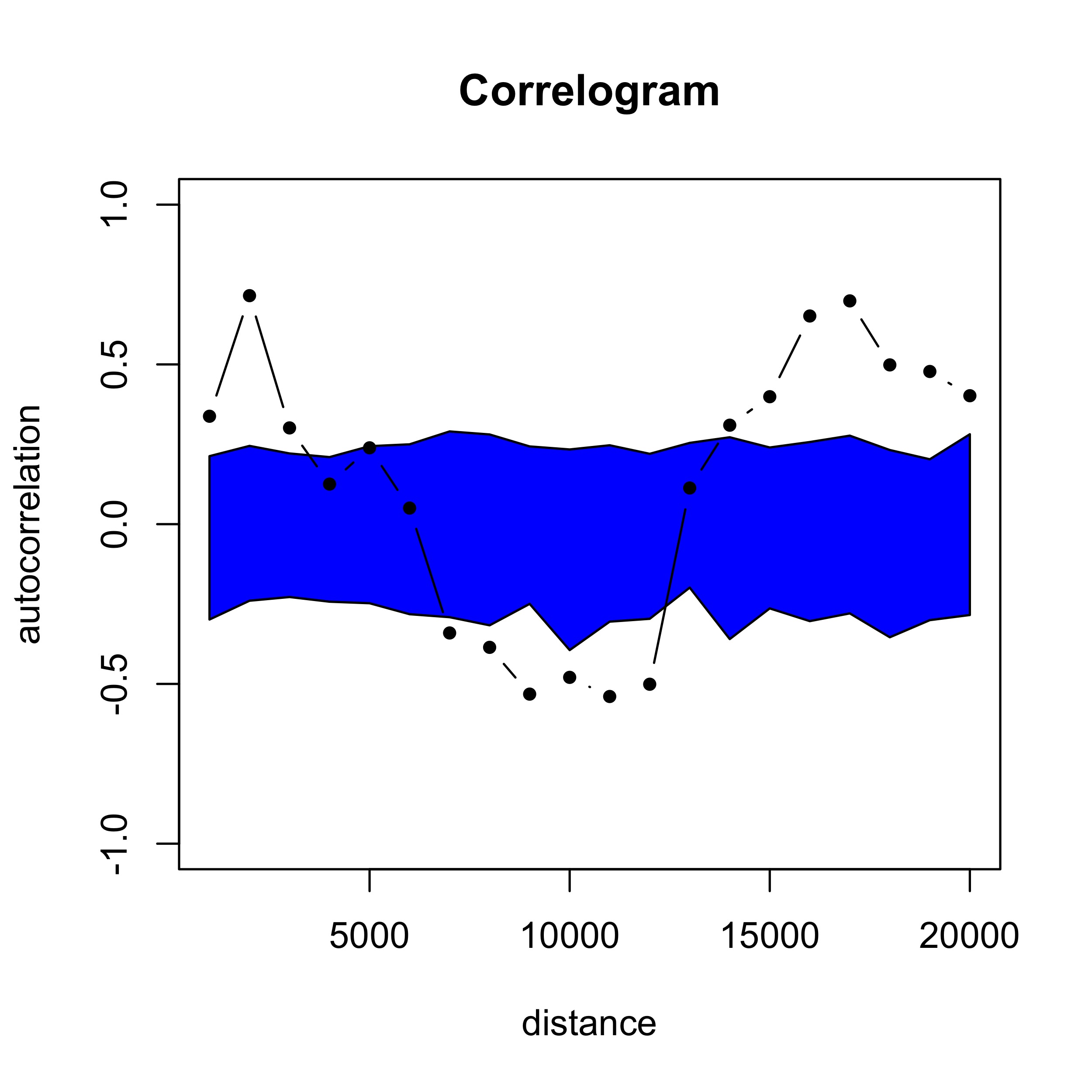


(c)


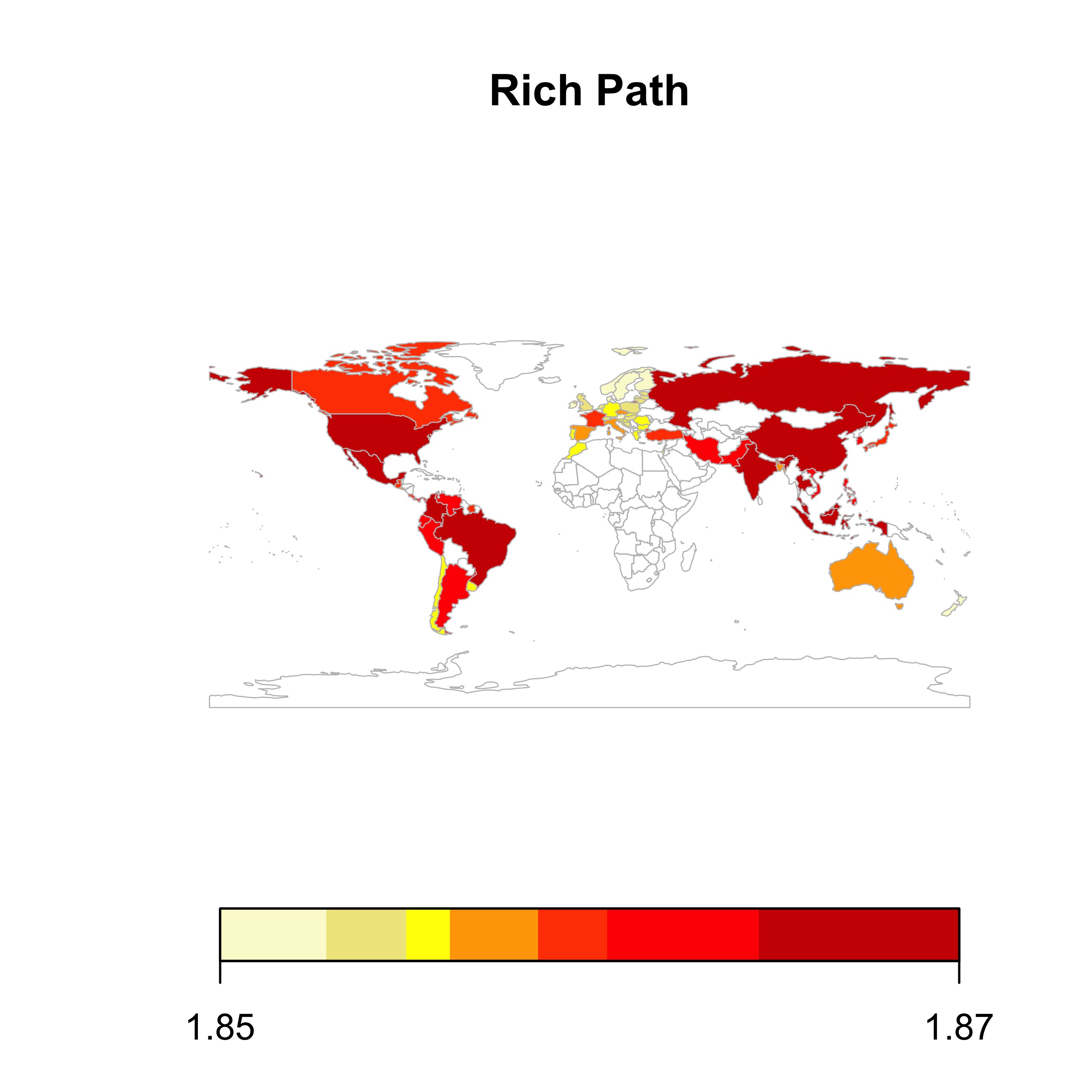


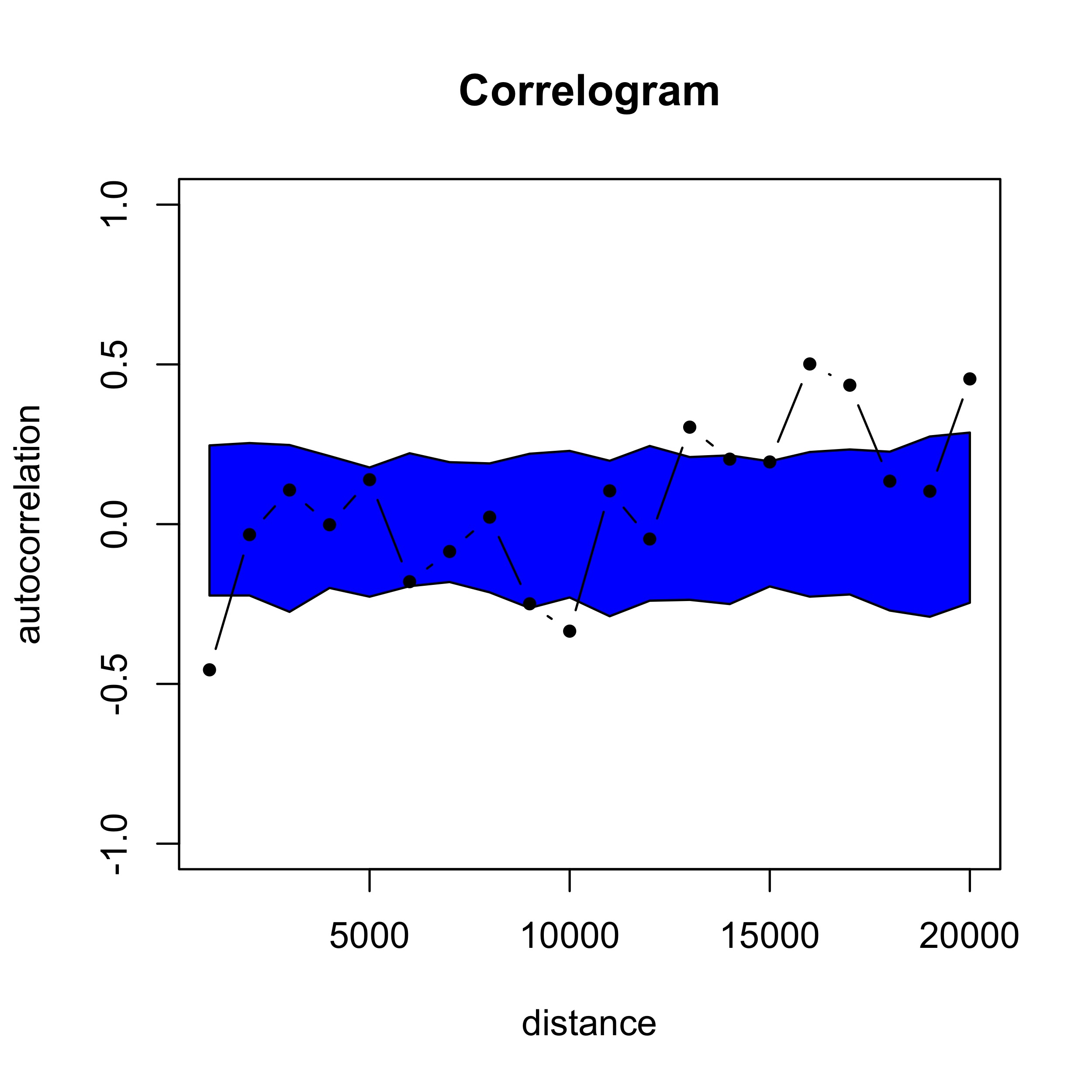


(d)


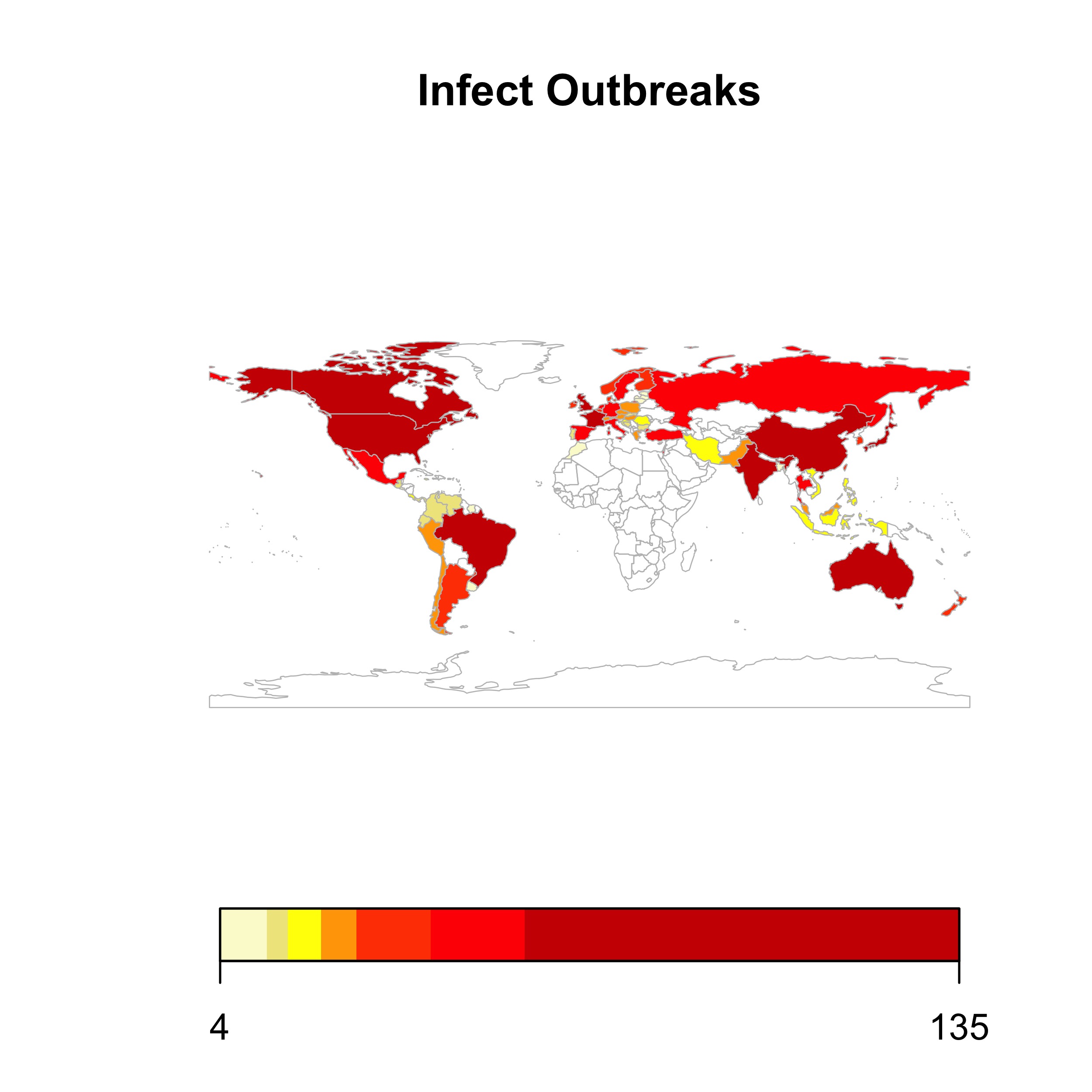


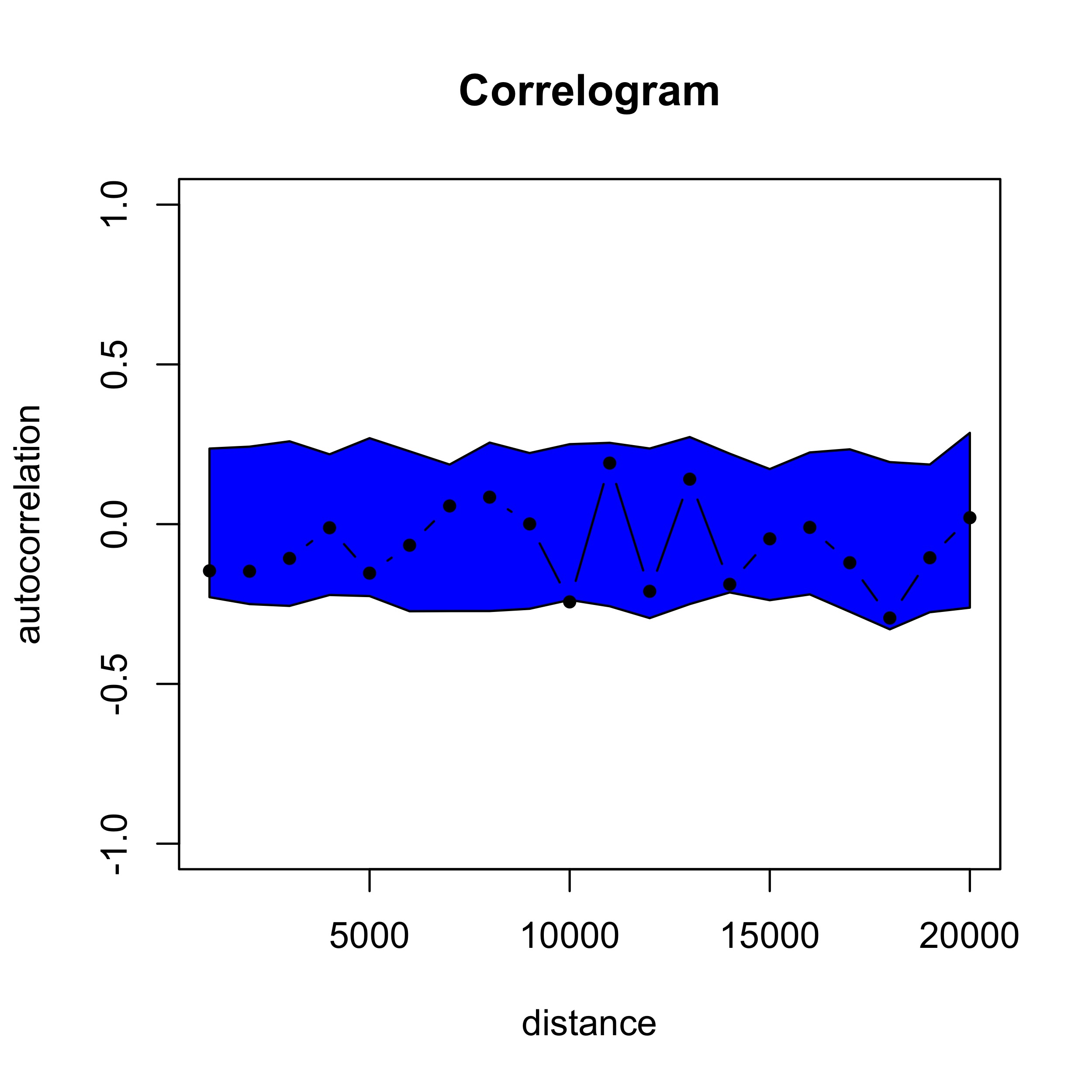


(e)


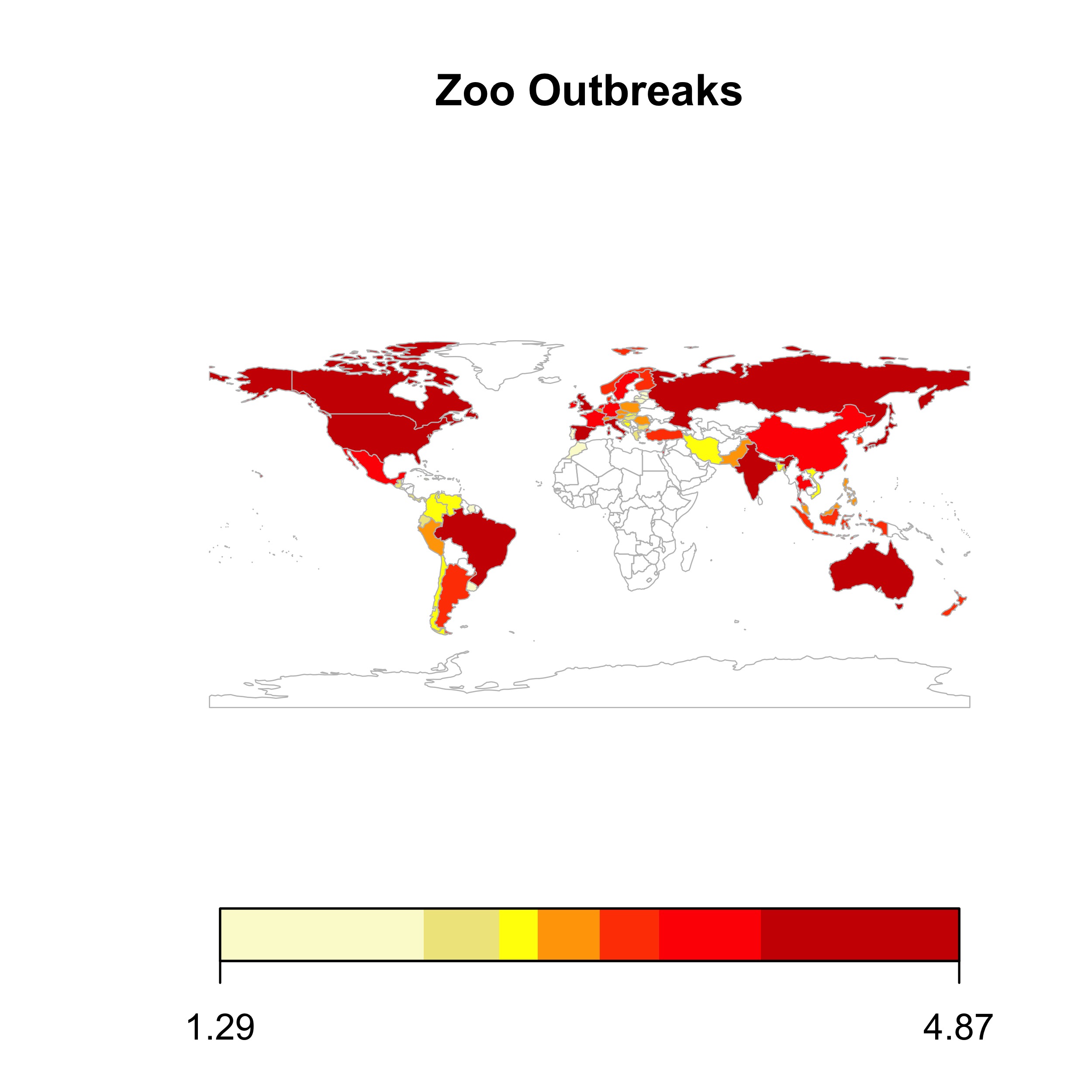


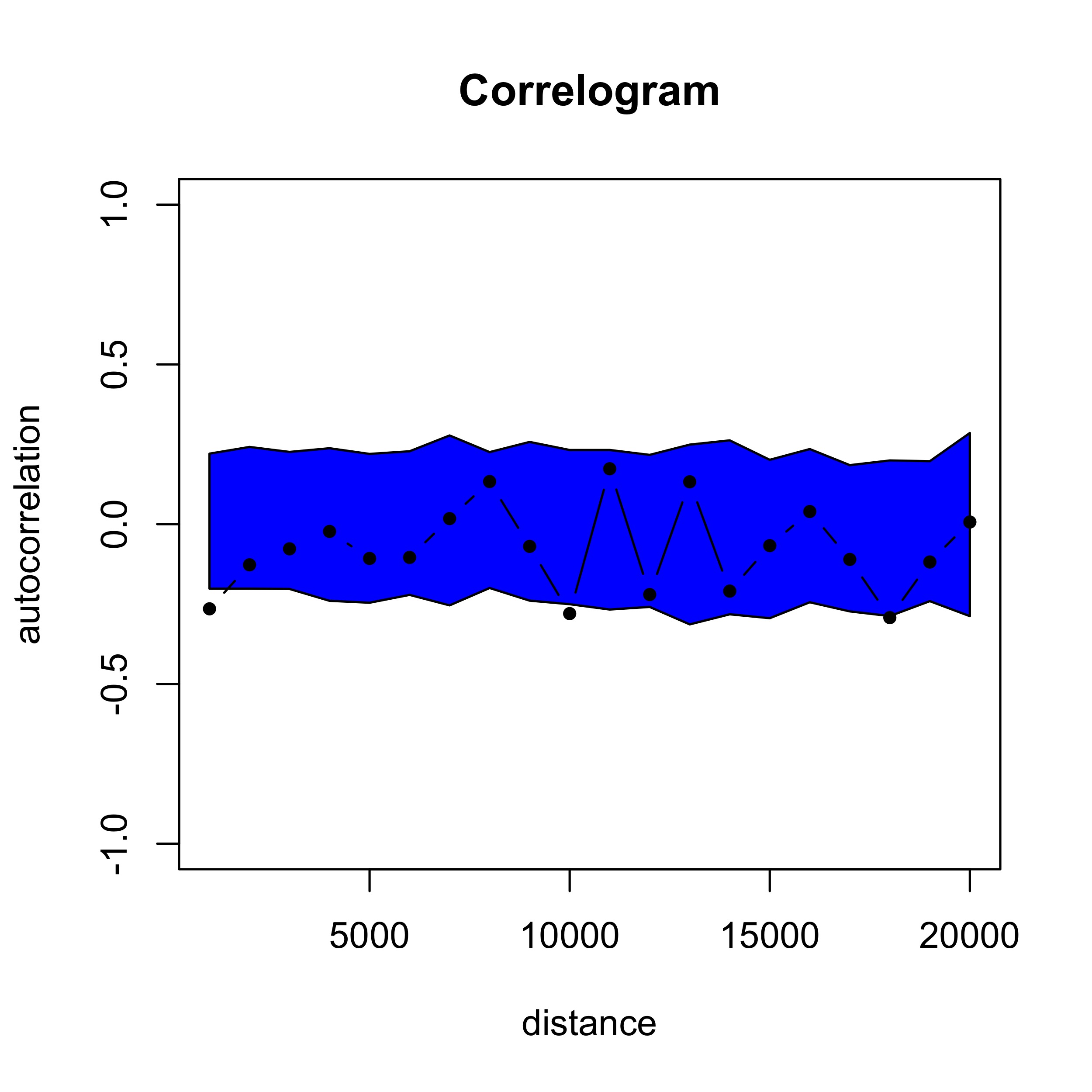


(f)


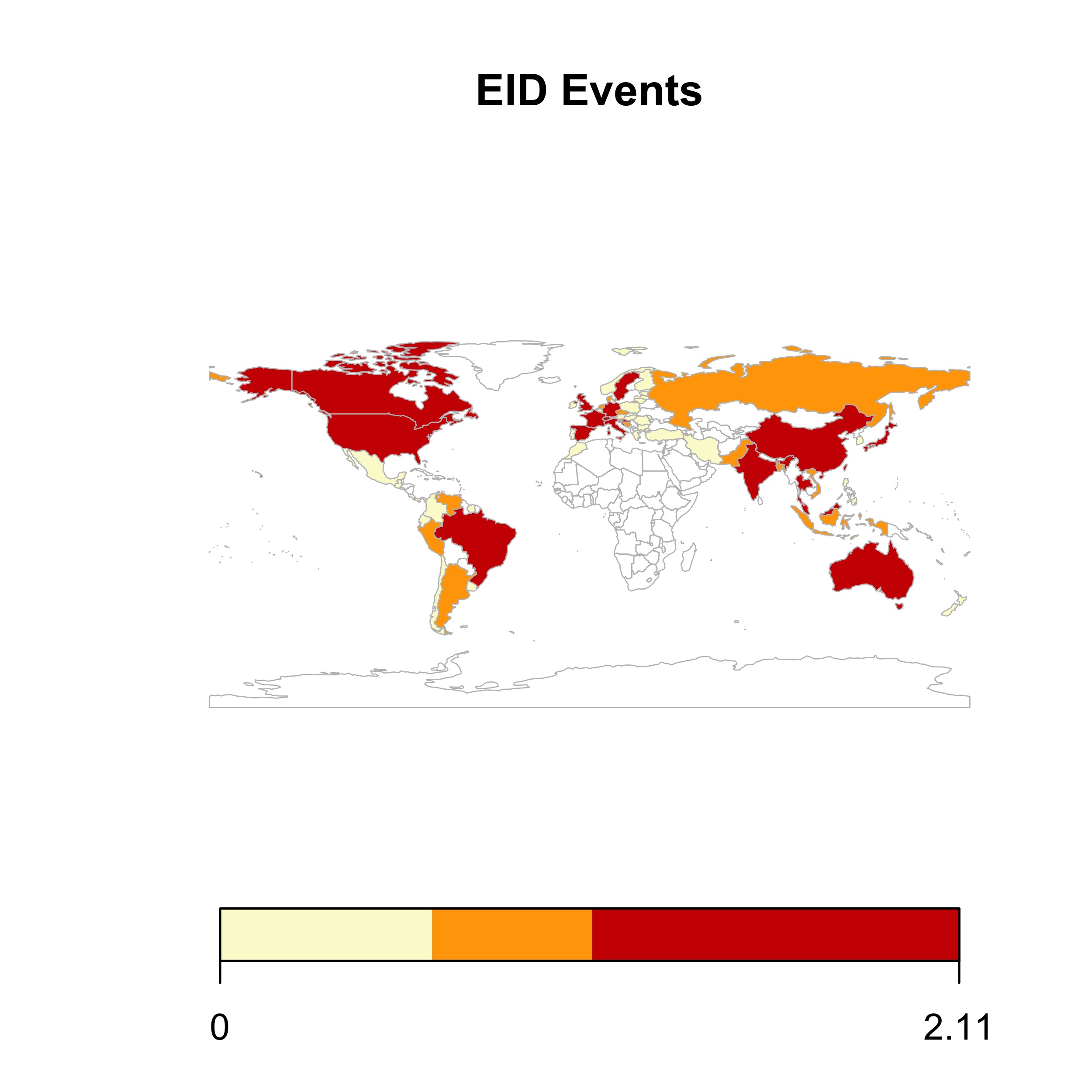


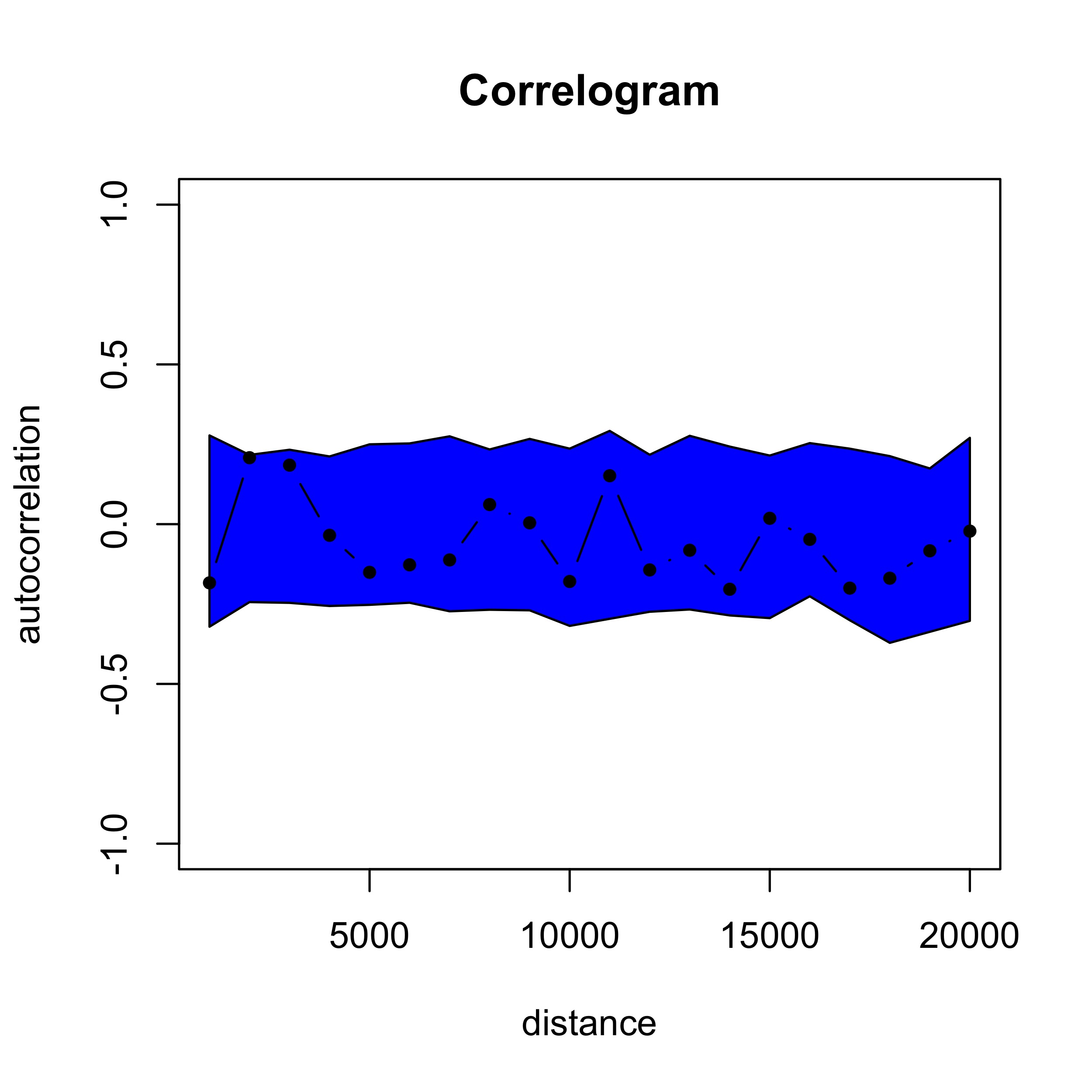


(g)


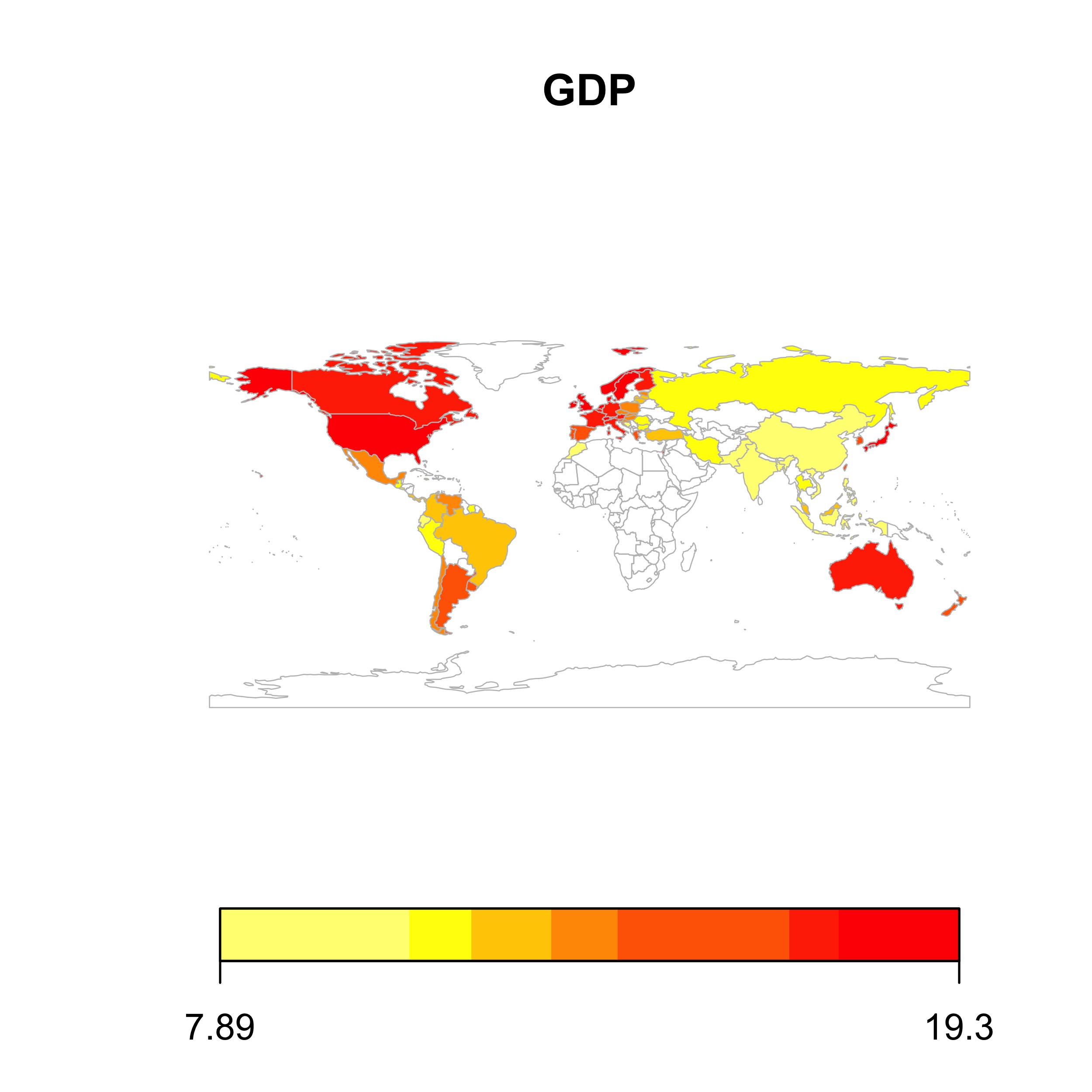


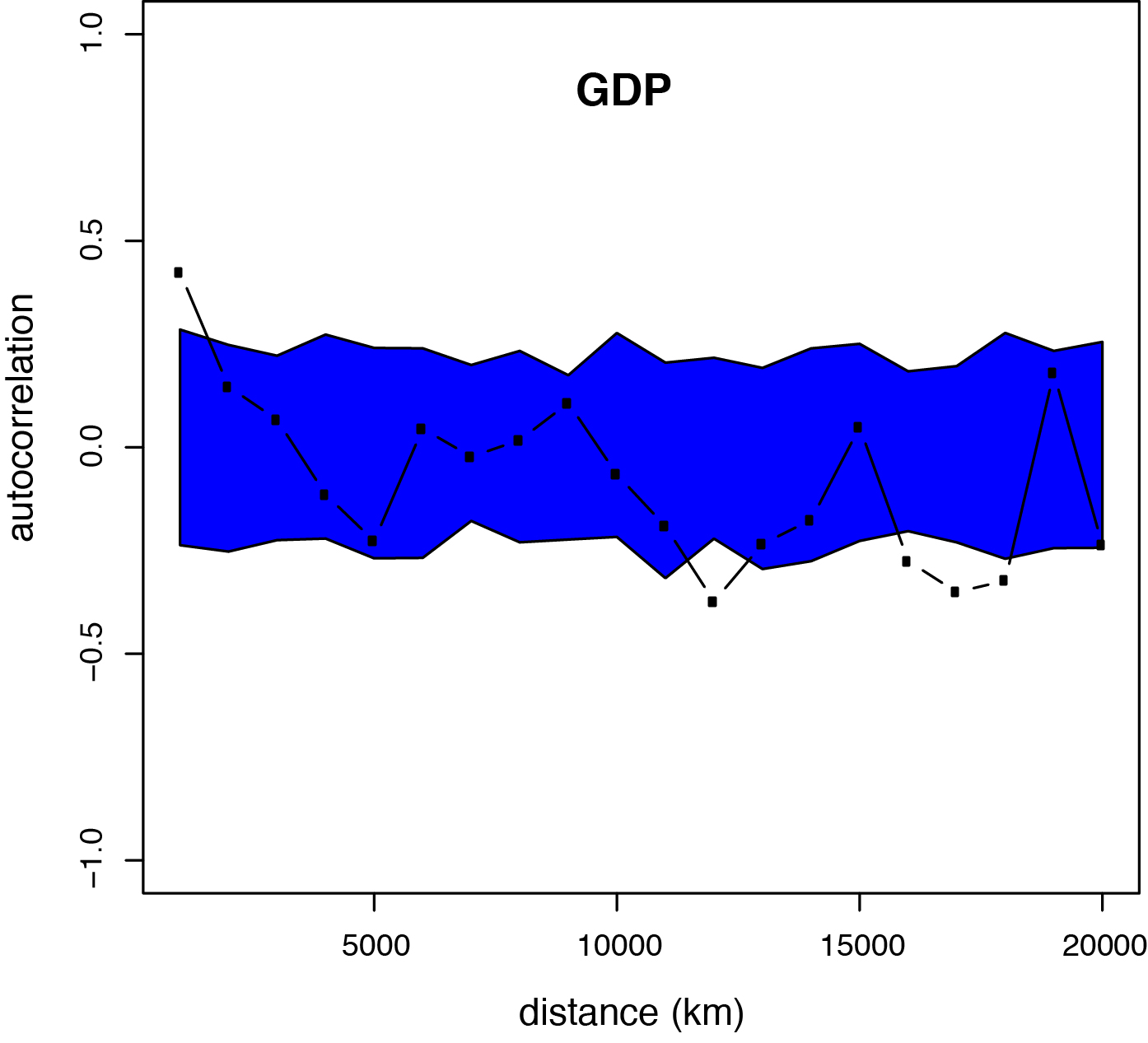

Supplement: Supplementary file 1 — Supplementary information [file 41598_2018_22014_MOESM1_ESM.doc]
